# Synthesis and Biological Evaluation of Novel 4-(4-Formamidophenylamino)-*N*-methylpicolinamide Derivatives as Potential Antitumor Agents

**DOI:** 10.3390/molecules26041150

**Published:** 2021-02-21

**Authors:** Nana Meng, Shuyan Zhou, Min Hu, Youzhi Xu, Yong Xia, Xiuxiu Zeng, Luoting Yu

**Affiliations:** 1 State Key Laboratory of Biotherapy and Cancer Center, West China Hospital, West China Medical School, Sichuan University and Collaborative Innovation Center, Chengdu 610041, China; mengnanain320@163.com (N.M.); 2018224065192@stu.scu.edu.cn (S.Z.); yzxu1997@163.com (Y.X.); yxia4@scu.edu.cn (Y.X.); xiuxiuwei@aliyun.com (X.Z.); 2 Hairong Research Institute, Sichuan Hairong Pharmaceutical Co., Ltd., Yangtze River Pharmaceutical Group, Chengdu 611830, China; 3 Open Laboratory, West China Institute of Women and Children’s Health, West China Second University Hospital, Sichuan University, Chengdu 610041, China; gym_w@163.com

**Keywords:** 4-(4-formamidophenylamino)-*N*-methylpicolinamide derivatives, anti-proliferation, angiogenesis inhibitors, apoptosis, pharmacology

## Abstract

A novel series of 4-(4-formamidophenylamino)-*N*-methylpicolinamide derivatives were synthesized and evaluated against different tumor cell lines. Experiments in vitro showed that these derivatives could inhibit the proliferation of two kinds of human cancer cell lines (HepG2, HCT116) at low micromolar concentrations and the most potent analog **5q** possessed broad-spectrum antiproliferative activity. Experiments in vivo demonstrated that **5q** could effectively prolong the longevity of colon carcinoma-burdened mice and slow down the progression of cancer cells by suppression of angiogenesis and the induction of apoptosis and necrosis.

## 1. Introduction

Cancer is one of the most common causes of death and its incidence is increasing worldwide [1]. Deregulated proliferation and inhibition of apoptosis lie at the heart of all tumor development, so they present two obvious targets for therapeutic intervention in all cancers [2,3]. In addition, angiogenesis plays a pivotal role in the progression of cancerous cells [4,5,6]. According to the opinion of Folkman, the growth and metastasis of neoplasms depend on new blood vessels. The diameter of neoplasms cannot exceed 2 mm without the support of new blood vessels. Therefore, the targeting of cancerous blood vessels is becoming a hot topic in the development of new drugs [7,8,9,10,11,12,13].

The findings mentioned above suggested that angiogenesis inhibitors and apoptosis inducers could have antitumor effects, and many angiogenesis inhibitors and apoptosis inducers were subsequently developed. Sorafenib, the first oral multikinase inhibitor to target Raf and that affects tumor signaling and the tumor vasculature, was approved by the FDA for the treatment of advanced renal cell carcinoma in December 2005 [14]. Axitinib, an oral angiogenesis inhibitor, was reported to be effective in a clinical trial [15]. Many other angiogenesis inhibitors were also developed, such as Enzastaurin [16], Sunitinib [17], Nilotinib [18], as well as Lapatinib [19]. Therefore, angiogenesis inhibitors and apoptosis inducers are promising agents in the exploitation and development of anticancer medicines.

Our laboratory has a long-standing interest in the design, synthesis, and biological evaluation of novel tumor growth inhibitors, angiogenesis inhibitors, and apoptosis inducers as potential new anticancer agents [20,21,22]. In a previous cell-based screening of anticancer drugs, we found an active structure: 4-(4-formamidophenylamino)-*N*-methyl picolinamide [23,24]. Based on this special structure, we designed and synthesized a series of new compounds (**5a**–**v**). These derivates were evaluated against different tumor cell lines by MTT assay. Experiments in vitro showed that most of the derivatives could inhibit the proliferation of two kinds of human cancer cell lines (HepG2, HCT116) at low micromolar concentrations in a dose-dependent manner. Preliminary structure–activity relationships were put forward based on the biological results. Compound **5q** was a promising agent, which significantly inhibited colon cancer growth in vivo with the suppression rate ranging from 70% to 90%. Inhibition of angiogenesis and apoptosis of cancer cells were also observed. The results suggest that **5q** is a potential new small-molecule antitumor agent in chemotherapy for colon carcinoma. Further structural optimization and mechanism studies are worth pursuing.

## 2. Results

### 2.1. Chemistry

**Scheme 1:**
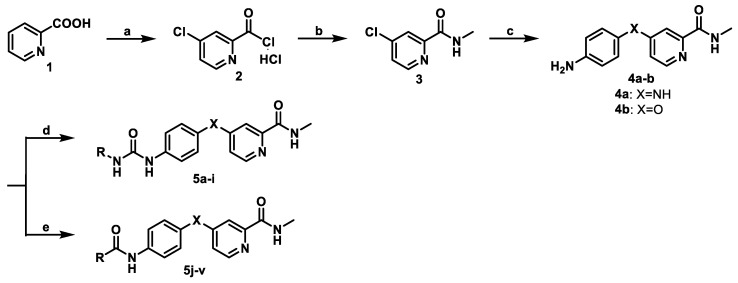
Synthesis of compounds **5a**–**5v**. Reagent and conditions: (a) chlorobenzene, sodium bromide, and thionyl chloride reflux for 19 h, 85 °C; (b) 25% methylamine aqueous, THF; (c) **4a**: (i) *N*-(4-aminophenyl)acetamide, 160 °C, (ii) EtOH, concentrated HCl, reflux for 4 h; **4b**: 4-aminophenol, sodium hydride, DMSO, 100 °C, stir for 3 h; (d) triphosgene, substituted aniline or cyclohexylamine, triethylamine, and CH_2_Cl_2_ reflux for 1 h; (e) K_2_CO_3_, THF, substituted benzoyl chloride, r.t.

The novel 4-(4-formamidophenylamino)-*N*-methylpicolinamide derivatives (**5a**–**v**) were prepared according to the general synthetic strategy illustrated in Scheme 1 [23,24]. One of the key intermediates, 4-(4-aminophenylamino)-*N*-methylpicolinamide **4a**, was prepared as follows: commercially available 2-picolinic acid was reacted with thionyl chloride and subsequently treated with methylamine to give **3**, which was heated with 4-amino-*N*-methylbenzamide at 160 °C with no solvent. The reaction mixture was dissolved in EtOH and concentrated HCl, then treated with 1% NaOH aqueous solution to yield **4a**. The other key intermediate **4b** was synthesized according to known methods [25,26,27]. With the key intermediates **4a** and **4b** in hand, derivatives **5a**–**i** were prepared through condensation of **4a** with aromatic isocyanates, which were easily gained via the treatment of substituted aniline or cyclohexylamine with triphosgene, in the presence of triethylamine. Using the same synthetic strategy, derivatives **5j**–**v** were prepared through acylation of **4a** or **4b** with a series of substituted benzoyl chloride compounds. The structures of **5a**–**v** were fully characterized by ^1^H-NMR, ^13^C-NMR, and ESI-MS analysis.

### 2.2. Pharmacology

#### 2.2.1. In Vitro Anticancer Activities

Anticancer activities of derivatives **5a**–**v** against HepG2 (Human Liver Cancer) and HCT116 (Human Colon Carcinoma) cell lines were determined by MTT assay, and the results expressed as IC_50_ are summarized in Table 1. As can be seen from the results, some derivatives (such as **5q**) exhibited potent antiproliferation activities as significant as Sorafenib against some cell lines. 

Replacement of the amide linkage with urea linkage yielded compounds **5a–i**. As shown in Table 1, the introduction of urea linkage to the benzene ring will slightly increase the inhibitory activity (compare **5a**–**i** with **5j**–**n**).

Based on the isostere principle, the NH group was replaced with oxygen, yielding compounds **5o**–**v**. As shown in Table 1, the introduction of a meta substituent on the phenyl ring such as lipophilic and bulky groups would strikingly increase inhibitory activity (**5q**) in vitro.

#### 2.2.2. In Vivo Antitumor Effects

**Figure 1:**
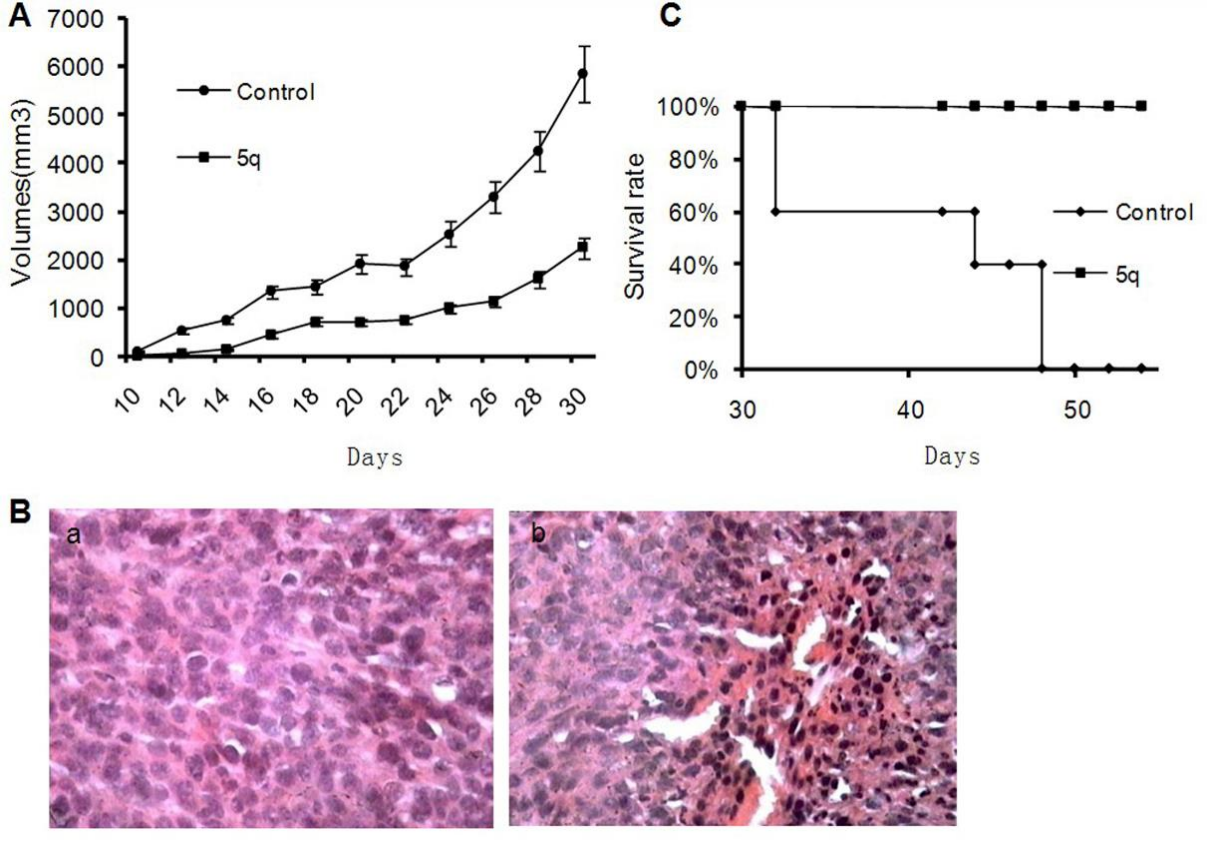
Effects of **5q** on tumor-bearing mice. **5q** was orally administered to Balb/c mice through gastric perfusion daily at a dosage of 75 mg/kg about 10 days after inoculation of cells. The volume of tumor was calculated by the following formula: volume (mm^3^) = long diameter (mm) × short diameter (mm)^2^ × 0.5236. (**A**) Oral treatment with **5q** presents a decrease in tumor volume against untreated (*p* < 0.05). (**B**) H&E staining of tumor tissue sections of untreated group (**a**), **5q**-treated group (**b**) (400×). (**C**) Oral treatment with **5q** presents a survival advantage against untreated. Data are plotted as the percentage of survival animal.

Based on the cytotoxic activities of the tested compounds in vitro, compound **5q** was chosen to investigate its growth inhibitory activity against the mice tumor model of colon cancer CT26 in Balb/c mice. When the tumor became palpable about 10 days after subcutaneous inoculation into the right flank of Balb/c mice, **5q** was administered orally once daily at a dosage of 75 mg/kg. The suppression of neoplasm was obvious in the curve because the average volumes of tumors in the **5q**-treated group were much smaller than that of the control group (*p* < 0.05) (Figure 1A). With the suppression rate ranging from 70% to 90%, the histological appearance of apoptotic and necrotic cells in the H.E. sections of the treated group verified the effectiveness of **5q** in the anticancer activity (Figure 1B). In particular, we observed that some neoplasms became black and sclerotic, finally resulting in shrinkage and exfoliation in the late stage of treatment, which suggested that large apoptosis and necrosis existed. Benefiting from the suppression of cell proliferation, the consequent prolongation of life span in the treated group was reasonable (Figure 1C).

#### 2.2.3. Apoptosis and Anti-Angiogenesis Effect

**Figure 2:**
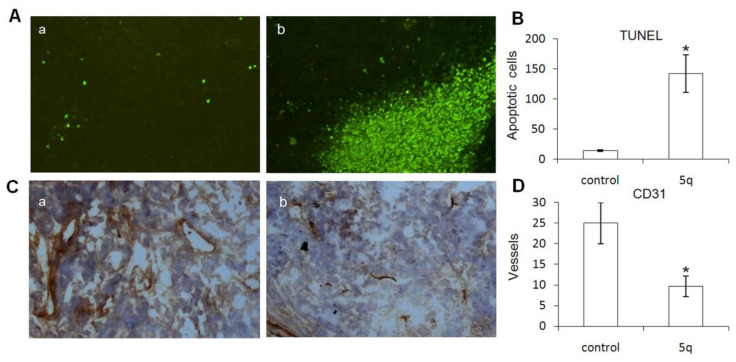
**5q** induces the apoptosis of cancer cells and inhibits angiogenesis. (**A**) **5q** induces the apoptosis of cancer cells. (**a**) Scattered positive signals in the control group. (**b**) Clustered positive signals in **5q**-treated group. (**B**) Quantitative analysis of the apoptotic cells. Data are represented as means ± standard deviation of cells per hpf (* *p* < 0.05) (200×). (**C**) **5q** inhibits angiogenesis. CD31 immunohistochemistry in the frozen sections from the control group (a) and **5q**-treated group (b). (**D**) Quantitative analysis of the vessel density. Data are represented as means ± standard deviation of vessels per hpf (* *p* < 0.05) (200×).

Observation of the pathology of the mice after 2 weeks treated with **5q** or control was conducted. The apoptosis of cancer cells was measured by TUNEL assay. Compared to the scattered positive signals in the control group, clustered positive signals in the **5q**-treated group strongly reflected the induction of apoptosis by the interference of **5q** (Figure 2A,B). Inhibition of angiogenesis was also observed. The vessel density was measured with CD31 immunohistochemistry in the frozen sections. More than a 50% decrease in vessel density could be seen in the **5q**-treated group compared to the control (Figure 2C,D).

## 3. Materials and Methods

### 3.1. Chemistry

All materials and reagents were obtained from commercial sources and were used without further purification unless stated. Melting points were determined on a SGW X-4 microscopic melting point (Shanghai Precision and Scientific Instrument Co., Ltd., Shanghai, China). ^1^H-NMR and ^13^C-NMR spectra were recorded on a Bruker Varian Unity Inova-400 (400/100 MHz) spectrometer (Bruker Corporation, Billerica, MA, USA) using TMS as the internal reference chemical. Shifts are expressed as δ values in ppm. Mass spectra were carried out on a triple-quadrupole mass spectrometry system from Waters Quattro PremierTM/XE (Waters Corporation, Milford, MA, USA). FT-IR spectra were recorded on a Nicolet 6700 FT-IR spectrometer (Thermo Fisher Scientific, Waltham, MA, USA).

#### 3.1.1. Synthesis of 4-Chloro-*N*-methylpicolinamide (**3**)

The compound was prepared according to the known method [25,26].

#### 3.1.2. Synthesis of 4-(4-Aminophenylamino)-*N*-methylpicolinamide (**4a**)

A mixture of 3 (10.4 g, 61.2 mmol) and 4-amino-*N*-methylbenzamide (9.01 g, 60 mmol) was heated at 160 °C for 1 h. The reaction mixture was dissolved in EtOH (228 mL) and concentrated HCl (45.7 mL) was added dropwise. The solution was stirred under reflux for 4 h and cooled to room temperature. The resulting solid was collected by filtration, washed with EtOH, and dried. The precipitate was suspended in 1% NaOH aqueous solution (385 mL) and stirred for 30 min. Then, the solid was collected by filtration, crystallized by EtOH, and decolorized with activated carbon to afford **4a** as a white solid (yield 91.2%). ^1^H-NMR (400 MHz, DMSO-*d*_6_) δ: 2.76 (3H, d, *J* = 4.4 Hz, CH_3_), 5.05 (2H, s, NH_2_), 6.59 (2H, d, *J* = 8.4 Hz, Ar-H), 6.71 (1H, dd, *J* = 5.6, 2.4 Hz, Ar-H), 6.87 (2H, d, *J* = 8.4 Hz, Ar-H), 7.31 (1H, d, *J* = 2.4 Hz, Ar-H), 8.07 (1H, d, *J* = 5.6 Hz, Ar-H), 8.50 (1H, s, Ar -NH-Ar), 8.57 ppm (1H, q, NHCH_3_). ^13^C-NMR (100 MHz, DMSO-*d*_6_) δ: 26.3, 106.2, 109.6, 115.1 (2C), 125.2 (2C), 128.4, 146.5, 149.0, 151.1, 154.2, 165.4 ppm. ESI-MS: *m*/*z* 243.26 [M + H^+^].

#### 3.1.3. Synthesis of 4-(4-Aminophenoxy)-*N*-methylpicolinamide (**4b**)

The compound was prepared according to the reported method [27].

#### 3.1.4. General Procedure for Synthesizing of **5a**–**i**

To a solution of triphosgene (5 mmol) in CH_2_Cl_2_ (10 mL), substituted aniline or cyclohexylamine (5.5 mmol) was added dropwise in CH_2_Cl_2_ (10 mL) followed by the drop wise addition of triethylamine (1.6 mL) in CH_2_Cl_2_ (10 mL). The mixture was stirred for 30 min. The solvent was removed on a rotary evaporator. The resulting residue was dissolved in CH_2_Cl_2_ (10 mL), and **4a** (5 mmol) in CH_2_Cl_2_ (10 mL) was added. After the mixture was refluxed for 1h and cooled to room temperature, the resulting solid was collected by filtration and crystallized by EtOH.

*N-methyl-4-(4-(3-phenylureido) phenylamino) picolinamide* (**5a**), Yield 63.2%; mp 176 °C; white solid; ^1^H-NMR (400 MHz, DMSO-*d*_6_) δ: 2.82 (3H, d, *J* = 4.8 Hz, CH_3_), 6.94–6.98 (1H, m, Ar-H), 7.08 (1H, q, Ar-H), 7.26–7.30 (4H, m, Ar-H), 7.47 (2H, d, *J* = 7.2 Hz, Ar-H), 7.52 (1H, d, *J* = 2.4 Hz, Ar-H), 7.59 (2H, d, *J* = 8.8 Hz, Ar-H), 8.18 (1H, d, *J* = 6.8 Hz, Ar-H), 9.22 (1H, s, NHCONH), 9.29 (1H, q, NHCH_3_), 9.42 (1H, s, NHCONH), 10.51 ppm (1H, br s, Ar-NH-Ar); ^13^C-NMR (100 MHz, DMSO-*d*_6_) δ: 26.3, 105.9, 109.8, 117.9 (2C), 118.9 (2C), 121.7, 124.2 (2C), 128.7 (2C), 130.7, 138.3, 139.8, 141.7, 143.5, 152.7, 156.4, 160.4 ppm; ESI-MS: *m*/*z* 360.21 [M − H^+^].

*N-methyl-4-(4-(3-(3-(trifluoromethyl) phenyl) ureido) phenylamino) picolinamide* (**5b**), Yield 85.2%; mp 186–190 °C; white solid; ^1^H-NMR (400 MHz, DMSO-*d*_6_) δ: 2.82 (3H, d, *J* = 4.0 Hz, CH3), 7.09 (1H, s, Ar-H), 7.30 (3H, d, *J* = 8.4 Hz, Ar-H), 7.50–7.54 (2H,m, Ar-H), 7.60(3H, d, *J* = 8.8 Hz, Ar-H), 8.01 (1H, s, Ar-H), 8.18 (1H, d, *J* = 6.8 Hz, Ar-H), 9.34 (1H, s, NHCH_3_), 9.64 (1H, s, NHCONH), 9.81 (1H, s, NHCONH), 10.65 ppm (1H, br s, Ar-NH-Ar); ^13^C-NMR (100 MHz, DMSO-*d*_6_) δ: 26.8, 56.5, 114.2, 114.2, 118.4, 119.6 (2C), 121.9, 123.3, 124.9 (2C), 126.0, 129.6, 129.9, 130.4, 131.1, 138.7, 141.2, 143.1, 153.2, 157.5, 160.2 ppm; ESI-MS: *m*/*z* 430.3 [M + H^+^].

*4-(4-(3-(3-fluorophenyl) ureido) phenylamino)-N-methylpicolinamide* (**5c**), Yield 80.3%; mp 224–226 °C; light green solid; ^1^H-NMR (400 MHz, DMSO-*d*_6_) δ: 2.8278 (3H, d, *J* = 4.0Hz, CH_3_), 6.76–6.80 (1H, m, Ar-H), 6.92 (1H, dd, *J* = 5.6, 2.4 Hz, Ar-H), 7.11–7.18 (3H, m, Ar-H), 7.28–7.33 (1H, m, Ar-H), 7.46–7.52 (4H, m, Ar-H), 8.17 (1H, d, *J* = 5.6 Hz, Ar-H), 8.62 (1H, q, NHCH_3_), 8.75 (1H, s, NHCONH), 8.91 (1H, s, NHCONH), 8.92 ppm (1H, br s, Ar-NH-Ar); ^13^C-NMR (100 MHz, DMSO-*d*_6_) δ: 25.8, 104.9, 106.4, 107.9, 109.7, 113.8, 119.6 (2C), 122.3 (2C), 130.2, 134.1, 135.4, 141.6, 148.7, 150.8, 152.3, 161.6, 163.2, 164.7 ppm; ESI-MS: *m*/*z* 378.2 [M − H^+^].

*4-(4-(3-(3-chlorophenyl) ureido) phenylamino)-N-methylpicolinamide* (**5d**), Yield 84.0%; mp 182 °C; yellow solid; ^1^H-NMR (400 MHz, DMSO-*d*_6_) δ: 2.79 (3H, d, *J* = 4.8 Hz, CH_3_), 6.97 (1H, dd, *J* = 6.0, 2.4 Hz, Ar-H), 6.99–7.02 (1H, m, Ar-H), 7.19 (2H, d, *J* = 8.8Hz, Ar-H), 7.26–7.32 (2H, m, Ar-H), 7.49–7.52 (3H, m, Ar-H), 7.72 (1H, d, *J* = 2.4 Hz, Ar-H), 8.17 (1H, d, *J* = 6.0 Hz, Ar-H), 8.83 (1H, q, NHCH_3_), 9.05 (1H, s, NHCONH), 9.15 (1H, s, NHCONH), 9.42 ppm (1H, br s, Ar-NH-Ar); ^13^C-NMR (100 MHz, DMSO-*d*_6_) δ: 19.0, 26.9, 56.5, 116.8, 117.6, 119.5 (2C), 121.8, 124.9 (2C), 130.9 (2C), 131.0, 133.7, 138.7, 141.8, 143.0, 153.0, 157.4, 160.2 ppm; ESI-MS: *m*/*z* 396.19 [M + H^+^].

*4-(4-(3-(3-bromophenyl) ureido) phenylamino) -N-methylpicolinamide* (**5e**), Yield 89.0%; mp 182 °C; yellow solid; ^1^H-NMR (400 MHz, DMSO-*d*_6_) δ: 2.82 (3H,d, *J* = 4.8 Hz, CH_3_), 7.00–7.02 (1H, m, Ar-H), 7.11 (1H, s, Ar-H), 7.29–7.33 (4H, m, Ar-H), 7.52 (1H, d, *J* = 2.8 Hz, Ar-H), 7.59 (2H,d, *J* = 8.8 Hz, Ar-H),7.71–7.72 (1H, m, Ar-H), 8.18 (1H, d, *J* = 6.8 Hz, Ar-H), 9.39 (1H, q, NHCH_3_), 9.63 (1H, s, NHCONH), 9.66 (1H, s, NHCONH), 10.74 ppm (1H, br s, Ar-NH-Ar); ^13^C-NMR (100 MHz, DMSO-*d*_6_) δ: 19.0, 26.9, 56.5, 116.7, 117.6,119.5 (2C), 121.8, 124.9 (2C), 130.9 (2C), 131.0, 133.7, 138.7, 141.8, 143.0, 153.0, 157.4, 160.2 ppm; ESI-MS: *m*/*z* 440.36 [M + H^+^].

*N-methyl-4-(4-(3-(3- nitrophenyl) ureido) phenylamino) picolinamide* (**5f**), Yield 91.0%; mp 220 °C; brown solid; ^1^H-NMR (400 MHz, DMSO-*d*_6_) δ: 2.82 (3H, d, *J* = 4.4 Hz, CH_3_), 7.12 (1H, q, Ar-H), 7.31 (2H, d, *J* = 8.8 Hz, Ar-H), 7.53 (1H, d, *J* = 2.4 Hz, Ar-H), 7.56 (1H, d, *J* = 8.0 Hz, Ar-H), 7.59–7.63 (2H, m, Ar-H), 7.71–7.74 (1H, m, Ar-H), 7.81–7.83 (1H, m, Ar-H), 8.18 (1H, d, *J* = 6.8 Hz, Ar-H), 8.56–8.58 (1H, m, Ar-H), 9.38 (1H, q, NHCH_3_), 9.74 (1H, s, NHCONH), 10.05 (1H, s, NHCONH), 10.75 ppm (1H, br s, Ar-NH-Ar); ^13^C-NMR (100 MHz, DMSO-*d*_6_) δ: 26.4, 111.7, 112.2, 116.2, 119.2 (2C), 123.9, 124.4 (2C), 130.1, 130.8, 131.3, 138.0, 141.1, 142.3, 148.1, 152.6, 156.8, 159.8, 160.4 ppm; ESI-MS: *m*/*z* 405.1 [M − H^+^].

*4-(4-(3-(3-methoxyphenyl) ureido) phenylamino)-N-methylpicolinamide* (**5g**), Yield 57.5%; mp176 °C; yellow solid; ^1^H-NMR (400 MHz, DMSO-*d*_6_) δ: 2.82 (3H, d, *J* = 4.4 Hz, CH_3_), 3.73 (3H, s, OCH_3_), 6.55 (1H, dd, *J* = 8.0, 2.4 Hz, Ar-H), 6.95 (1H, dd, *J* = 8.0, 1.2 Hz, Ar-H), 7.12–7.16 (1H, m, Ar-H), 7.19 (1H, d, *J* = 8.0 Hz, Ar-H), 7.21–7.22 (1H, m, Ar-H), 7.29 (2H, d, *J* = 8.8 Hz, Ar-H), 7.53 (1H, d, *J* = 2.4 Hz, Ar-H), 7.60 (2H, d, *J* = 8.8 Hz, Ar-H), 8.18 (1H, d, *J* = 6.8 Hz, Ar-H), 9.35 (1H, s, NHCONH), 9.39 (1H, q, NHCH3), 9.55 (1H, s, NHCONH), 10.75 ppm (1H, br s, Ar-NH-Ar); ^13^C-NMR (100 MHz, DMSO-*d*_6_) δ: 18.5, 26.4, 48.5, 54.9, 56.0, 103.7, 107.0, 110.3, 118.9 (2C), 124.4 (2C), 129.5, 130.3, 138.6, 141.0, 142.5, 152.6, 157.0, 159.6, 159.7 ppm; ESI-MS: *m*/*z* 390.21 [M + H^+^].

*4-(4-(3-(4-chloro-3-(trifluoromethyl) phenyl) ureido) phenylamino)-N-methylpicolinamide* (**5h**), Yield 92.3%; mp 223–225 °C; white solid; ^1^H-NMR (400 MHz, DMSO-*d*_6_) δ: 2.82 (3H, d, *J* = 4.4 Hz, CH_3_), 7.11 (1H, s, Ar-H), 7.31 (2H, d, *J* = 8.8 Hz, Ar-H), 7.53 (1H, d, *J* = 2.4 Hz, Ar-H), 7.59–7.67 (4H, m, Ar-H), 8.12 (1H, d, *J* = 2.4 Hz, Ar-H), 8.18 (1H, d, *J* = 6.8 Hz, Ar-H), 9.36 (1H, s, NHCH_3_), 9.66 (1H, s, NHCONH), 9.95 (1H, s, NHCONH), 10.70 ppm (1H, br s, Ar-NH-Ar); ^13^C-NMR (100 MHz, DMSO-*d*_6_) δ: 26.3,116.2, 116.2, 119.2 (2C), 121,4, 122.1, 122.5, 124.1, 124.4 (2C), 126.9, 130.8, 132.0, 138.0, 139.4, 140.7, 142.5, 152.5, 156.9, 159.7 ppm; ESI-MS: *m*/*z* 464.14 [M + H^+^].

*4-(4-(3-cyclohexylureido) phenylamino)-N-methylpicolinamide* (**5i**), Yield 70.5%; mp 236–240 °C; yellow solid; ^1^H-NMR (400 MHz, DMSO-*d*_6_) δ: 1.12–1.22 (3H, m, cyclohexane-H), 1.27–1.35 (2H, m, cyclohexane-H), 1.53–1.56 (1H, m, cyclohexane-H), 1.65–1.68 (2H, m, cyclohexane-H), 1.81 (2H, d, *J* = 9.6 Hz, cyclohexane-H), 2.78 (3H, d, *J* = 4.8 Hz, CH_3_), 3.46–3.47 (1H, m, cyclohexane-H), 6.05(1H, d, *J* = 7.6Hz, Ar-H), 6.86–6.87 (1H, m, Ar-H), 7.08 (2H, d, *J* = 8.4 Hz, Ar-H), 7.39 (2H, d, *J* = 8.4 Hz, Ar-H), 7.40 (1H, s, NHCONH), 8.14 (1H, d, *J* = 5.2 Hz, Ar-H), 8.31(1H, s, NHCONH), 8.61(1H, q, NHCH3), 8.81 ppm (1H, s, Ar-NH-Ar); ^13^C-NMR (100 MHz, DMSO-*d*_6_) δ: 24.8 (2C), 25.7, 26.3, 33.5 (2C), 48.1, 106.8, 110.0, 119.0 (2C), 123.1 (2C), 133.4, 137.3, 149.2, 151.2, 153.0, 154.9, 165.2 ppm; ESI-MS: *m*/*z* 366.3 [M − H^+^].

#### 3.1.5. General Procedure for Synthesizing of **5j**–**n**

To the suspension of anhydrous potassium carbonate (1.64 g, 12.5 mmol) and **4a** (1.22 g, 5 mmol) in THF (11.4 mL), substituted benzoyl chloride (7.5 mmol) was added dropwise. After being stirred at room temperature for 2 h, the mixture was extracted by ethyl acetate and water; then, the combined organic layers were dried over anhydrous Na_2_SO_4_ and concentrated under vacuum, and recrystallized by ethanol to give the resulting solids.

*N-methyl-4-(4-(3-(trifluoromethyl) benzamido) phenylamino) picolinamide* (**5j**), Yield 81.2%; mp 204–205 °C; white solid; ^1^H-NMR (400 MHz, DMSO-*d*_6_) δ: 2.79 (3H, d, *J* = 4.8 Hz, CH_3_), 6.99 (1H, dd, *J* = 5.2, 2.0 Hz, Ar-H), 7.23 (2H, d, *J* = 8.8 Hz, Ar-H), 7.55(1H, d, *J* = 1.2Hz, Ar-H), 7.78–7.81(3H, m, Ar-H), 7.97 (1H, m, Ar-H), 8.20 (1H, d, *J* = 5.6 Hz, Ar-H), 8.27–8.30 (2H, m, Ar-H), 8.63 (1H, q, NHCH_3_), 9.03 (1H, s, Ar-NH-Ar), 10.50 ppm (1H, s, CONH); ^13^C-NMR (100 MHz, DMSO-*d*_6_) δ: 26.3, 107.2, 110.5, 121.9 (2C), 122.1 (2C), 124.7, 124.7, 125.8, 128.5, 130.1, 132.2, 134.9, 136.3, 136.4, 149.4, 151.4, 152.4, 164.3, 165.2 ppm; ESI-MS: *m*/*z* 415.2 [M + H^+^].

*4-(4-(3- fluorobenzamido) phenylamino)-N-methylpicolinamide* (**5k**), Yield 51.2%; mp 216–218 °C; white solid; ^1^H-NMR (400 MHz, DMSO-*d*_6_) δ: 2.79 (3H, d, *J* = 4.8 Hz, CH_3_), 6.99 (1H, q, Ar-H), 7.22 (2H, d, *J* = 8.8 Hz, Ar-H), 7.44–7.48 (1H, m, Ar-H), 7.54 (1H, d, *J* = 1.6 Hz, Ar-H), 7.57–7.59 (1H, m, Ar-H), 7.77–7.83 (4H, m, Ar-H), 8.20 (1H, d, *J* = 5.6 Hz, Ar-H), 8.63 (1H, q, NHCH_3_), 9.01 (1H, s, Ar-NH-Ar), 10.34 ppm (1H, s, CONH); ^13^C-NMR (100 MHz, DMSO-*d*_6_) δ: 25.9, 106.7, 109.9, 121.4 (2C), 121.6 (2C), 130.5, 130.5, 134.5, 135.8, 137.2, 137.3, 148.9, 150.9, 151.9, 161.1, 162.7, 163.9, 164.7 ppm; ESI-MS: *m/z* 362.94 [M − H^+^].

*4-(4-(3- chlorobenzamido) phenylamino) -N-methylpicolinamide* (**5l**), Yield 50.2%; mp 202–204 °C; light green solid; ^1^H-NMR (400 MHz, DMSO-*d*_6_) δ: 2.79 (3H, d, *J* = 5.2 Hz, CH_3_), 6.99 (1H, dd, *J* = 5.6, 2.4 Hz, Ar-H), 7.22 (2H, d, *J* = 8.4 Hz, Ar-H), 7.54–7.60 (2H, m, Ar-H), 7.66–7.68 (1H, m, Ar-H),7.78 (2H, d, *J* = 8.4 Hz, Ar-H), 7.92 (1H, d, *J* = 8.0 Hz, Ar-H), 8.01 (1H, s, Ar-H), 8.20 (1H, d, *J* = 5.6 Hz, Ar-H), 8.64 (1H, q, NHCH_3_), 9.02 (1H, s, Ar-NH-Ar), 10.38 ppm (1H, s, CONH); ^13^C-NMR (100 MHz, DMSO-*d*_6_) δ: 25.9, 106.7, 109.9, 121.5 (2C), 121.6 (2C), 126.4, 127.3, 130.3, 131.3, 133.2, 134.5, 135.8, 136.9, 148.9, 150.8, 151.9, 163.8, 164.7 ppm; ESI-MS: *m/z* 381.15 [M + H^+^].

*N-methyl-4-(4- (3- nitrobenzamido) phenylamino) picolinamide* (**5m**), Yield 92.0%; mp 258–260 °C; yellow solid; ^1^H-NMR (400 MHz, DMSO-*d*_6_) δ: 2.79 (3H, d, *J* = 4.8 Hz, CH_3_),7.00 (1H, dd, *J* = 5.6, 2.4 Hz, Ar-H), 7.25 (2H, d, *J* = 8.8 Hz, Ar-H), 7.55 (1H, d, *J* = 2.4 Hz, Ar-H), 7.80 (2H, d, *J* = 8.8 Hz, Ar-H), 7.86 (1H, t, *J* = 8.0 Hz, Ar-H), 8.21 (1H, d, *J* = 5.6 Hz, Ar-H), 8.41–8.46 (2H, m, Ar-H), 8.64 (1H, q, NHCH_3_), 8.80 (1H, s, Ar-H), 9.04 (1H, s, Ar-NH-Ar), 10.62 ppm (1H, s, CONH); ^13^C-NMR (100 MHz, DMSO-*d*_6_) δ: 25.9, 106.8, 110.0, 121.4 (2C), 121.7 (2C), 122.3, 126.0, 130.1, 134.1, 134.2, 136.0, 136.3, 147.7, 148.9, 150.9, 151.8, 163.0, 164.7 ppm; ESI-MS: *m*/*z* 390.10 [M − H^+^].

*4-(4- (3-methoxybenzamido) phenylamino) -N-methylpicolinamide* (**5n**), Yield 86.0%; mp 169 °C; white solid; ^1^H-NMR (400 MHz, DMSO-*d*_6_) δ: 2.79 (3H, d, *J* = 4.8 Hz, CH3), 3.85 (3H, s, OCH_3_), 6.98 (1H, q, Ar-H), 7.15–7.18 (1H, m, Ar-H), 7.22 (2H, d, *J* = 8.8 Hz, Ar-H), 7.44–7.50 (2H, m, Ar-H), 7.54–7.56 (2H, m, Ar-H), 7.79 (2H, d, *J* = 8.8 Hz, Ar-H), 8.20 (1H, d, *J* = 5.6 Hz, Ar-H), 8.64 (1H, q, NHCH_3_), 9.01 (1H, s, Ar-NH-Ar), 10.25 ppm (1H, s, CONH); ^13^C-NMR (100 MHz, DMSO-*d*_6_) δ: 25.9, 55.3, 106.7, 109.9, 112.9, 117.2, 119.8, 121.5 (2C), 121.6 (2C), 129.5, 134.8, 135.6, 136.4, 148.9, 150.9, 151.9, 159.2, 174.7, 165.0 ppm; ESI-MS: *m*/*z* 375.14 [M − H^+^].

#### 3.1.6. General Procedure for Synthesizing of **5o**–**v**

To the suspension of anhydrous potassium carbonate (1.64 g, 12.5 mmol) and **4b** (1.22 g, 5 mmol) in THF (11.4 mL), substituted benzoyl chloride (7.5 mmol) was added dropwise. After being stirred at room temperature for 2 h, the mixture was extracted by ethyl acetate and water; then, the combined organic layers were dried over anhydrous Na_2_SO_4_ and concentrated under vacuum, and recrystallized by ethanol to give the resulting solids. In order to improve the solubility and bioavailability of compound **5q**, we prepared **5q·TsOH** by the following steps: To the suspension of **5q** (10.4 mmol) in ethyl acetate (47 mL) at 70 °C, *p*-toluenesulfonic acid (11.7 mmol) in a mixture of ethyl acetate (15 mL) and water (2 mL) was added dropwise. The solution was stirred at 70 ℃ for 2 h and cooled to room temperature. The resulting solid was collected by filtration, washed with ethyl acetate, and dried to afford **5q·TsOH** as a white solid.

*4-(4-benzamidophenoxy)-N-methylpicolinamide* (**5o**), Yield 78.1%; mp 180–182 °C; white solid; ^1^H-NMR (400 MHz, DMSO-*d*_6_) δ: 2.79 (3H, d, *J* = 4.8 Hz, CH_3_), 7.17 (1H, q, Ar-H), 7.22–7.26 (2H, m, Ar-H), 7.40 (1H, d, *J* = 2.8 Hz, Ar-H), 7.53–7.57 (2H, m, Ar-H), 7.59–7.64 (1H, m, Ar-H), 7.91–7.95 (2H, m, Ar-H), 7.96–7.99 (2H, m, Ar-H), 8.52 (1H, d, *J* = 5.6 Hz, Ar-H), 8.79 (1H, q, NHCH_3_), 10.42 ppm (1H, s, CONH); ^13^C-NMR (100 MHz, DMSO-*d*_6_) δ: 26.5, 109.3, 114.6, 121.8 (2C), 122.9 (2C), 122.9, 126.7, 130.7, 134.6, 136.6, 137.0, 148.2, 149.6, 150.8, 152.9, 163.8, 164.2, 166.2 ppm; ESI-MS: *m*/*z* 346.17 [M − H^+^].

*N-methyl-4-(4-(2-(trifluoromethyl) benzamido) phenoxy) picolinamide* (**5p**), Yield 92.0%; mp 178 °C; white solid; ^1^H-NMR (400 MHz, DMSO-*d*_6_) δ:2.79 (3H, d, *J* = 4.8 Hz, CH_3_), 7.17 (1H, dd, *J* = 5.6, 2.4 Hz, Ar-H), 7.24 (2H, d, *J* = 7.6Hz, Ar-H), 7.40 (1H, d, *J* = 2.4 Hz, Ar-H), 7.71–7.76 (2H, m, Ar-H), 7.80–7.88 (4H, m, Ar-H), 8.51 (1H, d, *J* = 5.6 Hz, Ar-H), 8.78 (1H, q, CONH), 10.73 ppm (1H, s, CONH); ^13^C-NMR (100 MHz, DMSO-*d*_6_) δ: 26.5 109.3, 114.5, 121.9 (2C), 122.9 (2C), 125.6, 126.2, 126.5, 129.0, 130.6, 133.1, 136.6, 137.2, 149.4, 150.9, 153.0, 164.2, 166.1, 166.3 ppm; ESI-MS: *m*/*z* 414.2 [M − H^+^].

*N-methyl-4-(4-(3-(trifluoromethyl) benzamido) phenoxy) picolinamide* (**5q**), Yield 95.0%; mp 152–153 °C; white solid; ^1^H-NMR (400 MHz, DMSO-*d*_6_) δ: 2.79 (3H, d, *J* = 4.8 Hz, CH_3_), 7.18 (1H, q, Ar-H), 7.26 (2H, d, *J* = 8.8 Hz, Ar-H), 7.40 (1H, d, *J* = 2.4 Hz, Ar-H), 7.79–7.83 (1H, m, Ar-H), 7.91 (2H, d, *J* = 9.2 Hz, Ar-H), 7.99 (1H, d, *J* = 8.0 Hz, Ar-H), 8.27–8.30 (2H, m, Ar-H), 8.52 (1H, d, *J* = 5.6 Hz, Ar-H), 8.79 (1H, q, NHCH_3_), 10.63 ppm (1H, s, CONH); ^13^C-NMR (100 MHz, DMSO-*d*_6_) δ: 26.4, 109.3, 114.6, 121.7 (2C), 122.8 (2C), 124.7, 124.8, 125.8, 128.6, 130.2, 132.3, 136.2, 137.1, 149.6, 150.8, 153.0, 164.2, 164.6, 166.3 ppm; ESI-MS: *m*/*z* 416.2 [M + H^+^].

*N-methyl-4-(4-(3-(trifluoromethyl) benzamido) phenoxy) picolinamide·p-toluenesul fonic acid* (**5q·TsOH**), Yield 95.0%; mp202–203 °C; white solid; ^1^H-NMR (400 MHz, DMSO-*d*_6_) δ: 2.29 (3H, s, Ar-CH3), 2.81 (3H, d, *J* = 4.4 Hz, N-CH_3_), 6.30 (1H, s, SO_3_H), 7.13 (2H, d, *J* = 7.6 Hz, Ar-H), 7.25–7.30 (3H, m Ar-H), 7.48–7.54 (3H, m, Ar-H), 7.80–7.84 (1H, m, Ar-H), 7.94 (2H, d, *J* = 8.8 Hz, Ar-H), 8.00 (1H, d, *J* = 8.0 Hz, Ar-H), 8.28–8.31 (2H, m, Ar-H), 8.57 (1H, d, *J* = 6.0 Hz, Ar-H), 8.94 (1H, d, *J* = 5.2 Hz, NHCH_3_), 10.66 ppm (1H, s, CONH); ^13^C-NMR (100 MHz, DMSO-*d*_6_) δ: 21.0, 26.6, 111.0, 114.9, 121.4 (2C), 122.7 (2C), 124.6, 125.8 (2C), 128.5 (2C), 129.0, 129.3, 129.6, 130.0, 132.1, 135.8, 137.6, 138.5, 145.1, 147.6, 148.4, 148.6, 161.1, 164.4, 169.1 ppm. ESI-MS: *m*/*z* 416.13 [M + H^+^]. IR 3341.6 cm^−1^, 3293.4 cm^−1^ (***v***_N-H_, CONH), 3107.5 cm^−1^, 3062.8 cm^−1^, 3032.9 cm^−1^ (***v***_ = C__-__H_, Ar-H), 2809.4 cm^−1^, 2717.2 cm^−1^ (***v***_N-H_, > N^+^H_2_), 1693.3 cm^−1^(***v*** as _N-C=O_, CONH), 1675.7 cm^−1^, 1547.1 cm^−1^ (***v***_N-C=O_, CONH), 1594.3 cm^−1^ (***v***_C=C_, Pyridine), 1615.8 cm^−1^, 1504.0 cm^−1^ (***v***_C=C_, phenyl), 1320.2 cm^−1^ (***v***_C-F_, ph-CF_3_), 1149.1 cm^−1^, 1123.6 cm^−1^ (***v***_C-F_, ph-CF3), 1194.3 cm^−1^ (***v***_S=O_, -SO_3_), 1032.3 cm^−1^, 1009.2 cm^−1^ (***v***_S-O_, -SO_3_), 847.9 cm^−1^, 824.5 cm^−1^, 700.0 cm^−1^ (δ_C-H_, Ar-H), 568.7 cm^−1^, 558.7 cm^−1^ (δ_C-F_,-CF_3_).

*N-methyl-4-(4-(4-(trifluoromethyl) benzamido) phenoxy) picolinamide* (**5r**), Yield 93.2%; mp 200 °C; white solid; ^1^H-NMR (400 MHz, DMSO-*d*_6_) δ: 2.79 (3H, d, *J* = 4.8 Hz, CH_3_), 7.18 (1H, dd, *J* = 5.6, 2.4 Hz, Ar-H), 7.26 (2H, d, *J* = 8.8 Hz, Ar-H), 7.41 (1H, d, *J* = 2.4 Hz, Ar-H), 7.92–7.95 (4H, m, Ar-H), 8.17 (2H, d, *J* = 7.6 Hz, Ar-H), 8.53 (1H, d, *J* = 5.6 Hz, Ar-H), 8.79 (1H, q, NHCH_3_), 10.63 ppm (1H, s, CONH); ^13^C-NMR (100 MHz, DMSO-*d*_6_) δ: 26.4, 109.3, 114.6, 121.8 (2C), 121.9 (2C), 125.7, 125.8, 126.4, 129.1, 131.1, 131.8, 137.1, 139.1, 149.5, 150.9, 153.0, 164.2, 164.9, 166.3 ppm; ESI-MS: *m*/*z* 414.2 [M − H^+^].

*4-(4-(3- fluorobenzamido) phenoxy)-N-methylpicolinamide* (**5s**), Yield 79.2%; mp 218–219 °C; white solid; ^1^H-NMR (400 MHz, DMSO-*d*_6_) δ: 2.79 (3H, d, *J* = 4.8 Hz, CH_3_), 7.18 (1H, dd, *J* = 5.6, 2.8 Hz, Ar-H), 7.23–7.27 (2H, m, Ar-H), 7.40 (1H, d, *J* = 2.8 Hz, Ar-H), 7.45–7.50 (1H, m, Ar-H), 7.59–7.64 (1H, m, Ar-H), 7.78–7.84 (2H, m, Ar-H), 7.90–7.94 (2H, m, Ar-H), 8.52 (1H, d, *J* = 5.6 Hz, Ar-H), 8.79 (1H, q, NHCH_3_), 10.48 ppm (1H, s, CONH); ^13^C-NMR (100 MHz, DMSO-*d*_6_) δ: 26.0, 108.8, 114.0, 121.2 (2C), 122.2 (2C), 123.9, 123.9, 130.5, 130.6, 136.7, 137.1, 137.1, 148.9, 150.3, 152.4, 163.7, 164.2, 165.8 ppm; ESI-MS: *m*/*z* 364.07 [M − H^+^].

*4-(4-(3- chlorobenzamido) phenoxy)-N-methylpicolinamide* (**5t**), Yield 75.1%; mp 168 °C; white solid; ^1^H-NMR (400 MHz, DMSO-*d*_6_) δ: 2.79 (3H, d, *J* = 4.8 Hz, CH_3_), 7.18 (1H, q, Ar-H), 7.25 (2H, d, *J* = 9.2 Hz, Ar-H), 7.40 (1H, d, *J* = 2.8 Hz, Ar-H), 7.57–7.61 (1H, m, Ar-H), 7.68–7.70 (1H, m, Ar-H), 7.90–7.95 (3H, m, Ar-H), 8.03 (1H, t, *J* = 2.0 Hz, Ar-H), 8.52 (1H, d, *J* = 5.6 Hz, Ar-H), 8.79 (1H, q, NHCH_3_), 10.51 ppm (1H, s, CONH); ^13^C-NMR (100 MHz, DMSO-*d*_6_) δ: 26.0, 108.8, 114.1, 121.2 (2C), 122.2 (2C), 126.5, 127.4, 130.4, 131.4, 133.2, 136.7, 136.8, 149.0, 150.4, 152.4, 163.7, 164.1, 165.8 ppm; ESI-MS: *m*/*z* 380.05 [M − H^+^].

*N-methyl-4-(4-(3-nitrobenzamido) phenoxy) picolinamide* (**5u**), Yield 75.3%; mp 204 °C; brown solid; ^1^H-NMR (400 MHz, DMSO-*d*_6_) δ: 2.79 (3H, d, *J* = 4.8 Hz, CH_3_), 7.19 (1H, dd, *J* = 5.6, 2.8 Hz, Ar-H), 7.26–7.30 (2H, m, Ar-H), 7.41 (1H, d, *J* = 2.4 Hz, Ar-H), 7.85–7.89 (1H, m, Ar-H), 7.91–7.95 (2H, m, Ar-H), 8.42–8.48 (2H, m, Ar-H), 8.53 (1H, d, *J* = 5.6 Hz), 8.78–8.82 (2H, m, Ar-H), 10.74 ppm (1H, s, CONH); ^13^C-NMR (100 MHz, DMSO-*d*_6_) δ: 26.5, 109.3, 114.6, 121.8 (2C), 122.9 (2C), 122.9, 126.7, 130.6, 134.6, 136.6, 137.0, 148.2, 149.6, 150.8, 152.9, 163.8, 164.2, 166.2 ppm; ESI-MS: *m*/*z* 391.07 [M − H^+^].

*4-(4-(cyclohexanecarboxamido) phenoxy)-N-methylpicolinamide* (**5v**), Yield 72.5%; mp 176–177 °C; white solid; ^1^H-NMR (400 MHz, DMSO-*d*_6_) δ: 1.18–1.33 (3H, m, cyclohexane-H), 1.38–1.47 (2H, m, cyclohexane-H), 1.66 (1H, d, *J* = 10.8 Hz, cyclohexane-H), 1.75–1.83 (4H, m, cyclohexane-H), 2.30–2.37 (1H, m, cyclohexane-H), 2.78 (3H, d, *J* = 4.8 Hz, CH_3_), 7.12–7.17 (3H, m, Ar-H), 7.36 (1H, d, *J* = 2.0 Hz, Ar-H), 7.71–7.75 (2H, m, Ar-H), 8.50 (1H, d, *J* = 5.6 Hz, Ar-H), 8.77 (1H, q, NHCH_3_), 9.97 ppm (1H, s, CONH); ^13^C-NMR (100 MHz, DMSO-*d*_6_) δ: 25.2 (2C), 25.4, 26.0 (2C), 29.1, 44.8, 108.7, 113.9, 120.8 (2C), 121.2 (2C), 137.3, 148.1, 150.3, 152.4, 163.7, 165.9, 174.3 ppm; ESI-MS: *m*/*z* 352.24 [M − H^+^].

### 3.2. Pharmacology

#### 3.2.1. Cell Culture

HepG2(ATCC*^®^* HB-8065™), HCT116(ATCC*^®^* CCL-247™), and CT26 (ATCC*^®^* CRL-2638™) cell lines were obtained from ATCC (Rockville, MD, USA). All experiments were performed in plastic flasks, dishes, and multi-well plates obtained from Costar, USA. Cells were cultured at 37 °C in a humidified atmosphere in the presence of 5% CO_2_. For routine maintenance, cells were cultured in 75 cm^2^ flasks containing RPMI medium 1640 or DMEM (Gibco BRL) supplemented with 10% (*v*/*v*) heat-inactivated fetal bovine serum, and containing standard concentrations of L-glutamine, penicillin, and streptomycin (Gibco BRL). Cells were harvested by trypsinization (0.05% (*w*/*v*) trypsin, 0.53 mM EDTA4Na) twice a week.

#### 3.2.2. MTT

Cellular survival was evaluated by the MTT method [28]. Briefly, cells (3000/well) were seeded in 96-well plates and cultured for 24 h, followed by treatment with the compounds for 48 h. Ten microliters of 10 mg/mL MTT was added per well and incubated for another 2 h at 37 °C. Then, the supernatant fluid was removed and 150 μL/well DMSO was added for 15–20 min. The absorbance (OD) of each well was measured at 570 nm, using a SpectraMAX M5 microplate spectrophotometer (Molecular Devices, Sunnyvale, CA, USA). The effect of compounds on tumor cell viability was expressed by the IC_50_ of each cell line.

#### 3.2.3. Mice Tumor Models and Treatment

The animal experiment was performed in female Balb/c mice (8 weeks of age) with six mice per group, provided by the Center of Experimental Animals, Sichuan University (Chengdu, China). They were treated and executed in accordance with the protocols approved by the Animal Use and Care Committee, West China Hospital, Sichuan University. When the tumor became palpable about 10 days after subcutaneous inoculation into the right flank of Blab/c mice, **5q** (10 mg/mL, dissolved in oil/water emulsion composed of 50% water, 45% peanut oil, 5% Tween80, and 0.5% CMC) was administered through gastric perfusion daily at a dosage of 75 mg/kg. Tumor diameters were measured once on another day. The volume of tumor was calculated by the following formula: volume (mm^3^) = long diameter (mm) × short diameter (mm)^2^ × 0.5236.

#### 3.2.4. Histological Analysis

Immunohistochemistry was performed as follows. Briefly, 4% paraformaldehyde-fixed, paraffin-embedded sections or frozen sections from tumor tissues were incubated with a monoclonal rabbit anti-mouse CD31, at a 1:300 to 1:100 dilution (Santa Cruz) at 4 °C overnight. Following washes in PBS, the secondary antibody, biotinylated goat anti-rabbit antibody at a 1:1000 dilution (Vector), was added. The sections were then stained with streptavidin biotin reagents (Vector). Vessel density was determined by counting the number of microvessels per high-power field in the section with an antibody reactive to CD31. The TUNEL Kit (Promega) is based on the enzymatic addition of digoxigenin-nucleotide to the nicked DNA by terminal deoxynucleotidyl transferase. Sections in HE staining and immunohistochemical staining were observed by two pathologists in a blinded manner.

## 4. Conclusions

In summary, a series of new 4-(4-formamidophenylamino)-*N*-methylpicolinamide derivatives were synthesized and evaluated for their biological activities. Experiments in vitro showed that most of the derivatives could inhibit the proliferation of two kinds of human cancer cell lines (HepG2, HCT116) at low micromolar concentrations in a dose-dependent manner. Preliminary structure–activity relationships were put forward based on the biological results. Compound **5q** was a promising agent, which significantly inhibited colon cancer growth in vivo with the suppression rate ranging from 70% to 90%. Inhibition of angiogenesis and apoptosis of cancer cells were also observed. The results suggest that **5q** is a potential new small-molecule antitumor agent in chemotherapy for colon carcinoma. Further structural optimization and mechanism studies are worth pursuing.

## Figures and Tables

**Table 1:**
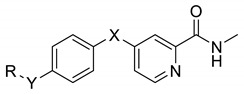
The antiproliferation activities of compounds **5a**–**v** against HepG2 and HCT116.

Compound	X	Y	R	IC_50_(μM) ^1^	
				HepG2	HCT116
**5a**	NH	NHCONH	Ph-	48.4 ± 2.5	28.2 ± 1.6
**5b**	NH	NHCONH	3-CF_3_-Ph-	16.8 ± 2.0	14.9 ± 1.1
**5c**	NH	NHCONH	3-F-Ph-	40.0 ± 3.1	18.1 ± 1.0
**5d**	NH	NHCONH	3-Cl-Ph-	22.4 ± 1.4	21.0 ± 1.1
**5e**	NH	NHCONH	3-Br-Ph-	18.0 ± 1.2	17.2 ± 1.2
**5f**	NH	NHCONH	3-NO_2_-Ph-	24.1 ± 1.3	27.6 ± 1.6
**5g**	NH	NHCONH	3-MeO-Ph-	27.6 ± 1.7	17.1 ± 0.8
**5h**	NH	NHCONH	4-Cl-3-CF_3_-Ph-	25.2 ± 1.3	29.2 ± 1.3
**5i**	NH	NHCONH	cyclohexane-	17.3 ± 1.0	12.1 ± 0.7
**5j**	NH	CONH	3-CF_3_-Ph-	16.8 ± 1.1	31.5 ± 1.9
**5k**	NH	CONH	3-F-Ph-	60.2 ± 3.4	17.9 ± 1.2
**5l**	NH	CONH	3-Cl-Ph-	94.4 ± 3.9	25.6 ± 1.4
**5m**	NH	CONH	3-NO_2_-Ph-	>100	>100
**5n**	NH	CONH	3-MeO-Ph-	32.2 ± 2.0	29.2 ± 1.9
**5o**	O	CONH	Ph-	55.1 ± 3.2	61.6 ± 3.1
**5p**	O	CONH	2-CF_3_-Ph-	>100	98.0 ± 4.2
**5q**	O	CONH	3-CF_3_-Ph-	6.6 ± 0.3	5.3 ± 0.3
**5r**	O	CONH	4-CF_3_-Ph-	51.4 ± 2.8	48.8 ± 3.2
**5s**	O	CONH	3-F-Ph-	>100	56.2 ± 3.8
**5t**	O	CONH	3-Cl-Ph-	68.2 ± 31	30.6 ± 2.8
**5u**	O	CONH	3-NO_2_-Ph-	>100	>100
**5v**	O	CONH	cyclohexane-	>100	55.8 ± 4.1
Sorafenib ^2^				15.3 ± 0.8	13.1 ± 0.7

^1^ The cytotoxicity effects of various compounds were determined by the MTT assay, and the results were expressed as the mean IC_50_ calculated from three independent experiments. ^2^ We select Sorafenib as the reference compound based on its structural similarity.

## Data Availability

The data presented in this study are available in supplementary materials.

## Supplementary Materials

The following are available online. ^1^H-NMR, ^13^C-NMR, FT-IR and ESI-MS spectra of **5q·TsOH** are available online. ^1^H-NMR spectra of **5a**–**v** and **4a** are also provided as Supplementary Materials.

## Sample Availability

Samples of the compounds **5a–5v** are not available from the authors.

## References

[1] Torre L.A., Siegel R.L., Ward E.M., Jemal A. (2016). Global Cancer Incidence and Mortality Rates and Trends-An Update. Cancer Epidemiol. Biomark. Prev..

[2] Evan G.I., Vousden K.H. (2001). Proliferation, cell cycle and apoptosis in cancer. Nature.

[3] Viallard C., Larrivee B. (2017). Tumor angiogenesis and vascular normalization: Alternative therapeutic targets. Angiogenesis.

[4] Sajib S., Zahra F.T., Lionakis M.S., German N.A., Mikelis C.M. (2018). Mechanisms of angiogenesis in microbe-regulated inflammatory and neoplastic conditions. Angiogenesis.

[5] Ferguson F.M., Gray N.S. (2018). Kinase inhibitors: The road ahead. Nat. Rev. Drug Discov..

[6] Du Z., Lovly C.M. (2018). Mechanisms of receptor tyrosine kinase activation in cancer. Mol. Cancer.

[7] Folkman J., Long D.M., Becker F.F. (1963). Growth and metastasis of tumor in organ culture. Cancer.

[8] Ferrara N., Kerbel R.S. (2005). Angiogenesis as a therapeutic target. Nature.

[9] Dai Y., Hartandi K., Ji Z., Ahmed A.A., Albert D.H., Bauch J.L., Bouska J.J., Bousquet P.F., Cunha G.A., Glaser K.B. (2007). Discovery of N-(4-(3-amino-1H-indazol-4-yl)phenyl)-N’-(2-fluoro-5-methylphenyl)urea (ABT-869), a 3-aminoindazole-based orally active multitargeted receptor tyrosine kinase inhibitor. J. Med. Chem..

[10] Sun L., Liang C., Shirazian S., Zhou Y., Miller T., Cui J., Fukuda J.Y., Chu J.Y., Nematalla A., Wang X. (2003). Discovery of 5-[5-fluoro-2-oxo-1,2- dihydroindol-(3Z)-ylidenemethyl]-2,4- dimethyl-1H-pyrrole-3-carboxylic acid (2-diethylaminoethyl)amide, a novel tyrosine kinase inhibitor targeting vascular endothelial and platelet-derived growth factor receptor tyrosine kinase. J. Med. Chem..

[11] Pietras K., Rubin K., Sjoblom T., Buchdunger E., Sjoquist M., Heldin C.H., Ostman A. (2002). Inhibition of PDGF receptor signaling in tumor stroma enhances antitumor effect of chemotherapy. Cancer Res..

[12] Potashman M.H., Bready J., Coxon A., DeMelfi T.M., DiPietro L., Doerr N., Elbaum D., Estrada J., Gallant P., Germain J. (2007). Design, synthesis, and evaluation of orally active benzimidazoles and benzoxazoles as vascular endothelial growth factor-2 receptor tyrosine kinase inhibitors. J. Med. Chem..

[13] Lindahl P., Johansson B.R., Leveen P., Betsholtz C. (1997). Pericyte loss and microaneurysm formation in PDGF-B-deficient mice. Science.

[14] Wilhelm S., Carter C., Lynch M., Lowinger T., Dumas J., Smith R.A., Schwartz B., Simantov R., Kelley S. (2006). Discovery and development of sorafenib: A multikinase inhibitor for treating cancer. Nat. Rev. Drug. Discov..

[15] Spano J.P., Chodkiewicz C., Maurel J., Wong R., Wasan H., Barone C., Letourneau R., Bajetta E., Pithavala Y., Bycott P. (2008). Efficacy of gemcitabine plus axitinib compared with gemcitabine alone in patients with advanced pancreatic cancer: An open-label randomised phase II study. Lancet.

[16] Oh Y., Herbst R.S., Burris H., Cleverly A., Musib L., Lahn M., Bepler G. (2008). Enzastaurin, an oral serine/threonine kinase inhibitor, as second- or third-line therapy of non-small-cell lung cancer. J. Clin. Oncol..

[17] Demetri G.D., van Oosterom A.T., Garrett C.R., Blackstein M.E., Shah M.H., Verweij J., McArthur G., Judson I.R., Heinrich M.C., Morgan J.A. (2006). Efficacy and safety of sunitinib in patients with advanced gastrointestinal stromal tumour after failure of imatinib: A randomised controlled trial. Lancet.

[18] Deremer D.L., Ustun C., Natarajan K. (2008). Nilotinib: A second-generation tyrosine kinase inhibitor for the treatment of chronic myelogenous leukemia. Clin. Ther..

[19] Medina P.J., Goodin S. (2008). Lapatinib: A dual inhibitor of human epidermal growth factor receptor tyrosine kinases. Clin. Ther..

[20] Zeng X.X., Zheng R.L., Zhou T., He H.Y., Liu J.Y., Zheng Y., Tong A.P., Xiang M.L., Song X.R., Yang S.Y. (2010). Novel thienopyridine derivatives as specific anti-hepatocellular carcinoma (HCC) agents: Synthesis, preliminary structure-activity relationships, and in vitro biological evaluation. Bioorg. Med. Chem. Lett..

[21] Wen J., Fu A.F., Chen L.J., Xie X.J., Yang G.L., Chen X.C., Wang Y.S., Li J., Chen P., Tang M.H. (2009). Liposomal honokiol inhibits VEGF-D-induced lymphangiogenesis and metastasis in xenograft tumor model. J. Cancer.

[22] Luo Y., Xu Y., Chen L., Hu J., Peng C., Xie D., Shi J., Huang W., Xu G., Peng M. (2009). Semi-synthesis and anti-proliferative activity evaluation of novel analogues of Honokiol. Bioorg. Med. Chem. Lett..

[23] Yu L., Zhao Y., Wei Y., Yang S., Yang L. (2009). 4-(4-aminoanilino)-2-(methylcarbamoyl) Pyridine and Its Derivatives, and Their Preparation Method and Application.

[24] Yu L., Zhao Y., Wei Y., Yang S., Yang L. (2009). 4-(4-benzoylaminophenoxy)-2-(methylcarbamoyl) Pyridine and Its Derivatives, and Their Preparation Method and Application.

[25] Anchoori R.K., Kortenhorst M.S., Hidalgo M., Sarkar T., Hallur G., Bai R., Diest P.J., Hamel E., Khan S.R. (2008). Novel microtubule-interacting phenoxy pyridine and phenyl sulfanyl pyridine analogues for cancer therapy. J. Med. Chem..

[26] Michael L., Reinhold G., Oliver K., Mike M., Klaus M., Matthias M.G., Jurjen S., Mathias B., Jana L., Wernner H. (2006). Procede de Preparation de 4-{4-[({[4-chloro-3-(trifluoromethyl)phenyl]amino}carbonyl)amino]phenoxy}-n-methylpyridine-2-carboxa-mide.

[27] Bankston D., Dumas J., Natero R., Riedl B., Monahan M.K., Sibley R. (2002). A scaleable synthesis of BAY 43-9006: A potent Raf kinase inhibitor for the treatment of cancer. Org. Process. Res. Dev..

[28] Cao Z., Zhen R., Lin H., Luo S., Zhou Y., Xu Y., Zeng X., Wang Z., Zhou L., Mao Y. (2011). SKLB610: A Novel Potential Inhibitor of Vascular Endothelial Growth Factor Receptor Tyrosine Kinases Inhibits Angiogenesis and Tumor Growth in Vivo. Cell. Physiol. Biochem..

