# Supplementary material for: Synthesis and Biological Evaluation of Novel 4-(4-Formamidophenylamino)-N-methylpicolinamide Derivatives as Potential Antitumor Agents
Source: Molecules. 2021 Feb 21;26(4):1150. doi: 10.3390/molecules26041150 (PMC7926825; doi:10.3390/molecules26041150)

Supplementary Materials

Synthesis and biological evaluation of novel 4-(4-formamidophenylamino)-*N*-methylpicolinamide derivatives as potential antitumor agents

Nana Meng [1,2,#], Shuyan Zhou [1,#], Min Hu[3], Youzhi Xu [1], Yong Xia [1], Xiuxiu Zeng [1] and Luoting Yu [1,*]

[1] State Key Laboratory of Biotherapy and Cancer Center, West China Hospital, West China Medical School, Sichuan University and Collaborative Innovation Center, Chengdu, Sichuan 610041, P. R. China; mengnanain320@163.com(Nana Meng);15250963890@163.com(Shuyan Zhou); 2513925609@qq.com(Min Hu); 273107786@qq.com(Youzhi Xu); 335265685@qq.com(Yong Xia);315263230@qq.com(Xiuxiu Zeng);yuluot@scu.edu.cn(Luoting Yu);

[2] Sichuan Hairong Pharmaceutical Co.,Ltd, Yangtze River Pharmaceutical Group, Chengdu 611830, Sichuan, China;

[3] Open Laboratory, West China Institute of Women and Children's Health, West China Second University Hospital, Sichuan University, Chengdu, Sichuan 610041, P. R. China;

* Correspondence: yuluot@scu.edu.cn; Tel.: +86-28-85164063; Fax: +86-28-85164060

[#] These authors contribute equally.

Contents

1、^{1}H and ^{13}C NMR, FT-IR and ESI-MS spectra of 5q•TsOH 3
1.1 ^{1}H NMR of 5q•TsOH 3
1.2 ^{13}C NMR of 5q•TsOH 4
1.3 FT-IR of 5q•TsOH 5
1.4 ESI-MS of 5q•TsOH 6
2、^{1}H NMR of 5a-v and 4a 7
2.1 ^{1}H NMR of 5a 7
2.2 ^{1}H NMR of 5b 8
2.3 ^{1}H NMR of 5d 9
2.4 ^{1}H NMR of 5e 10
2.5 ^{1}H NMR of 5f 11
2.6 ^{1}H NMR of 5g 12
2.7 ^{1}H NMR of 5h 13
2.8 ^{1}H NMR of 5j 14
2.9 ^{1}H NMR of 5k 15
2.10 ^{1}H NMR of 5l 16
2.11 ^{1}H NMR of 5m 17
2.12 ^{1}H NMR of 5n 18
2.13 ^{1}H NMR of 5o 19
2.14 ^{1}H NMR of 5q 20
2.15 ^{1}H NMR of 5s 21
2.16 ^{1}H NMR of 5t 22
2.17 ^{1}H NMR of 5u 23
2.18 ^{1}H NMR of 5v 24
2.19 ^{1}H NMR of 4a 26

1、^{1}H and ^{13}C NMR, FT-IR and ESI-MS spectra of 5q•TsOH

1.1 ^{1}H NMR of 5q•TsOH

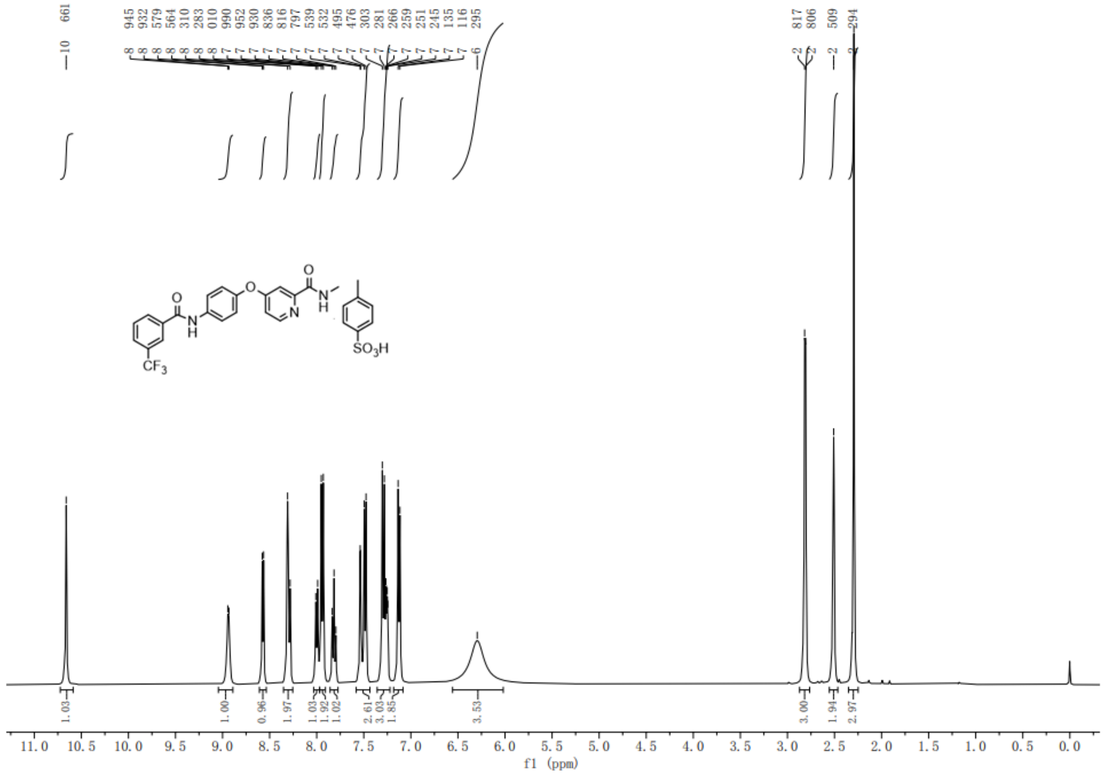

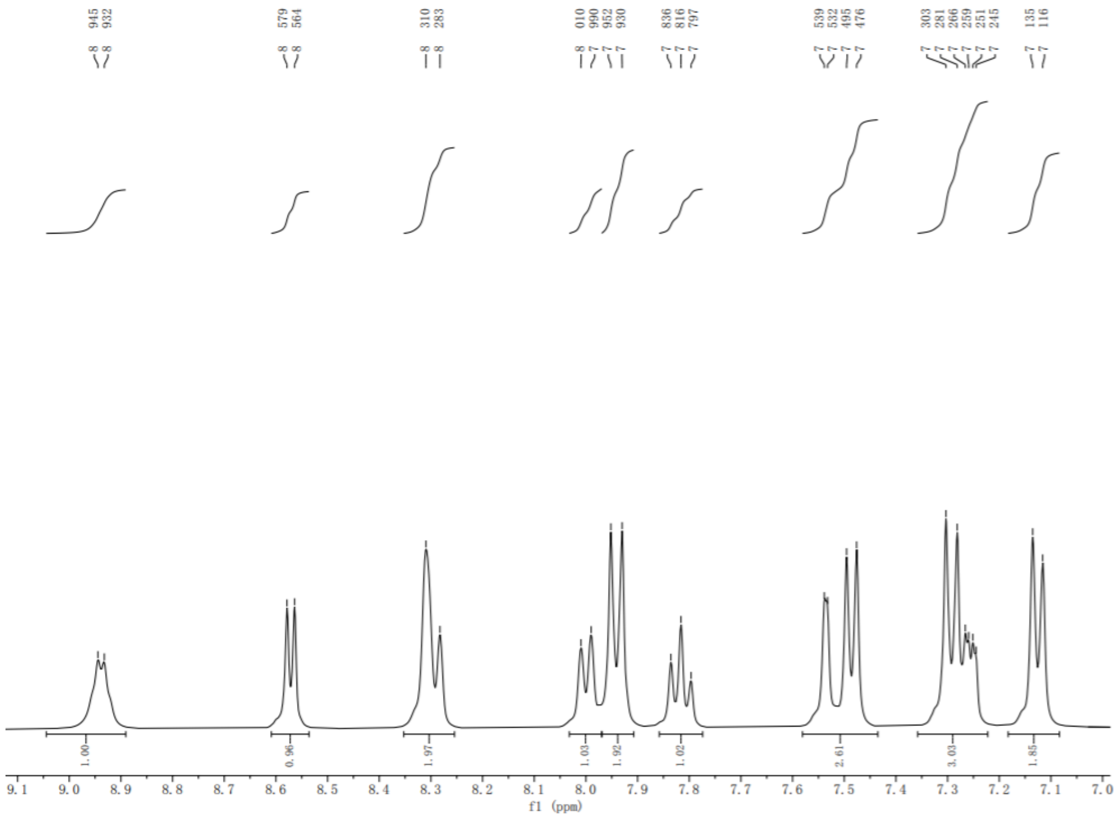

1.2 ^{13}C NMR of 5q•TsOH

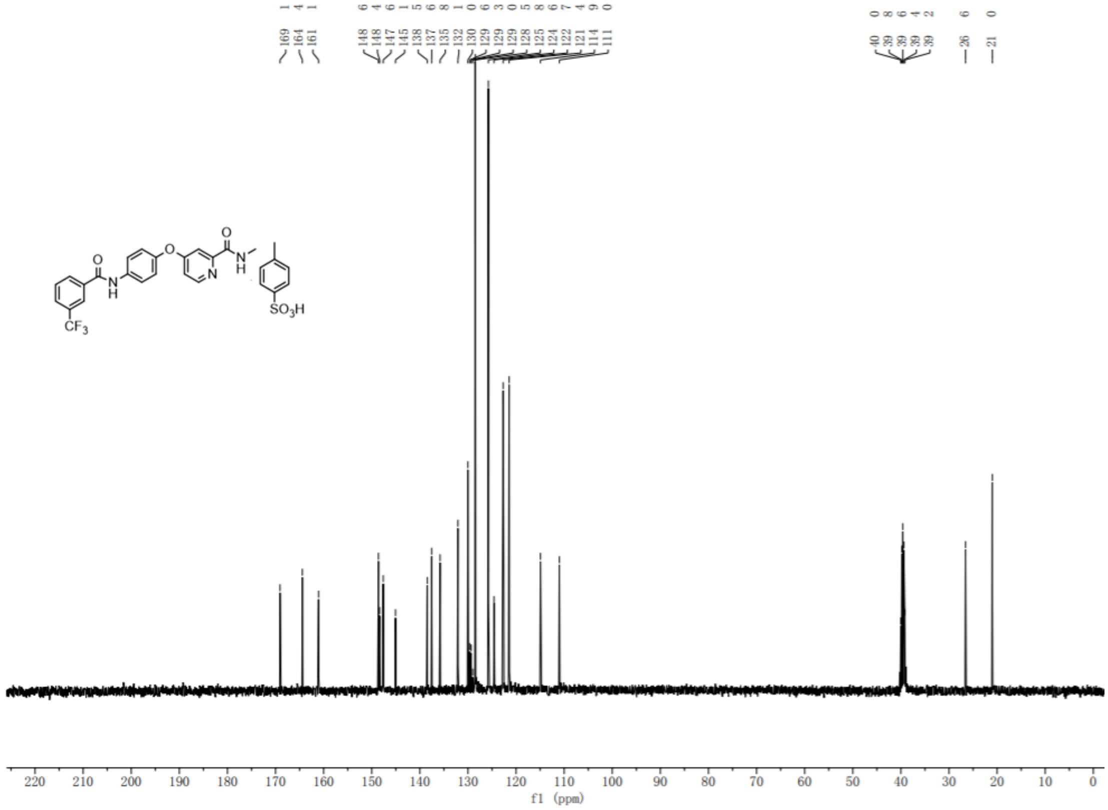

1.3 FT-IR of 5q•TsOH

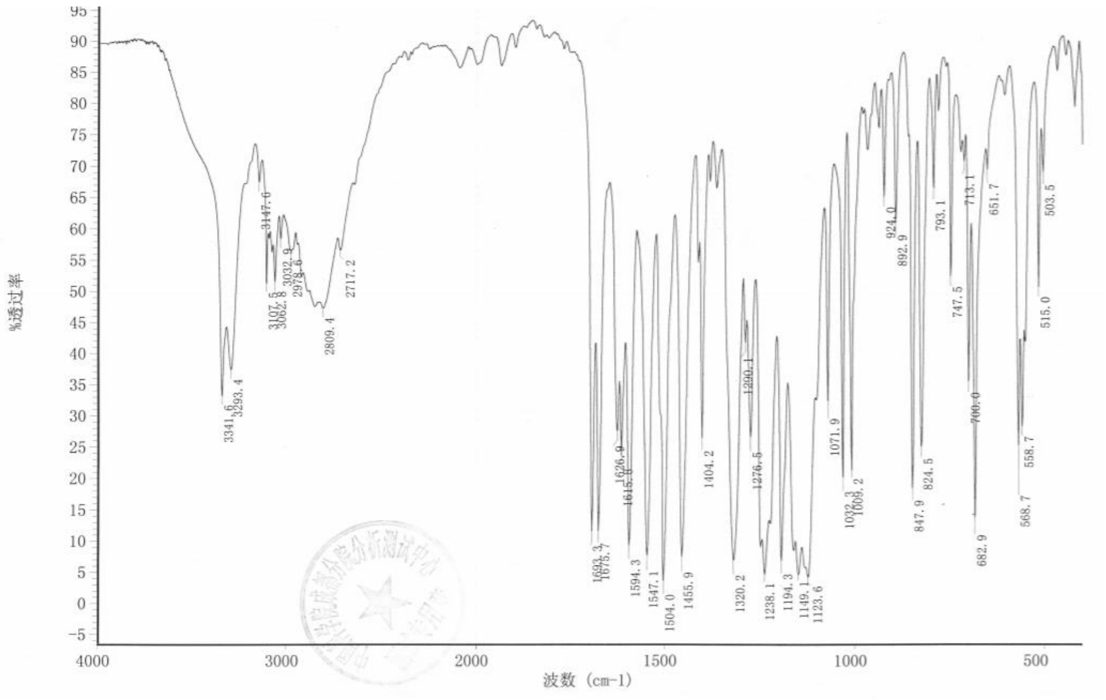

1.4 ESI-MS of 5q•TsOH

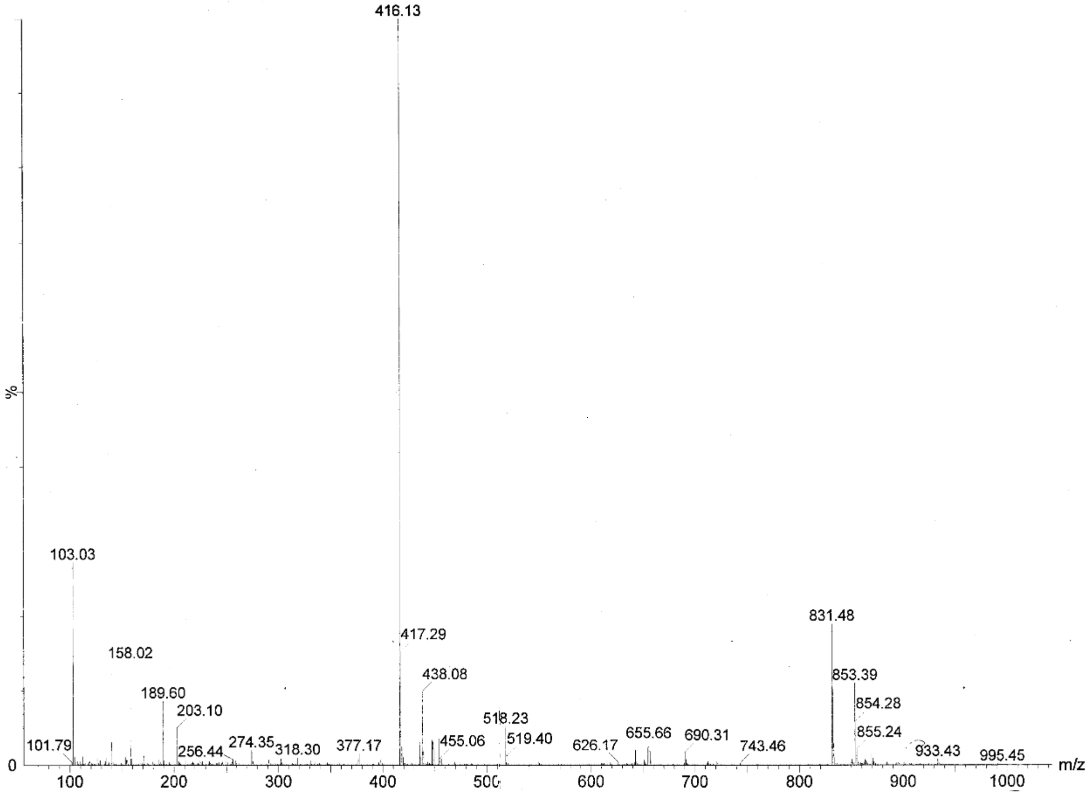

2、^{1}H NMR of 5a-v and 4a

2.1 ^{1}H NMR of 5a

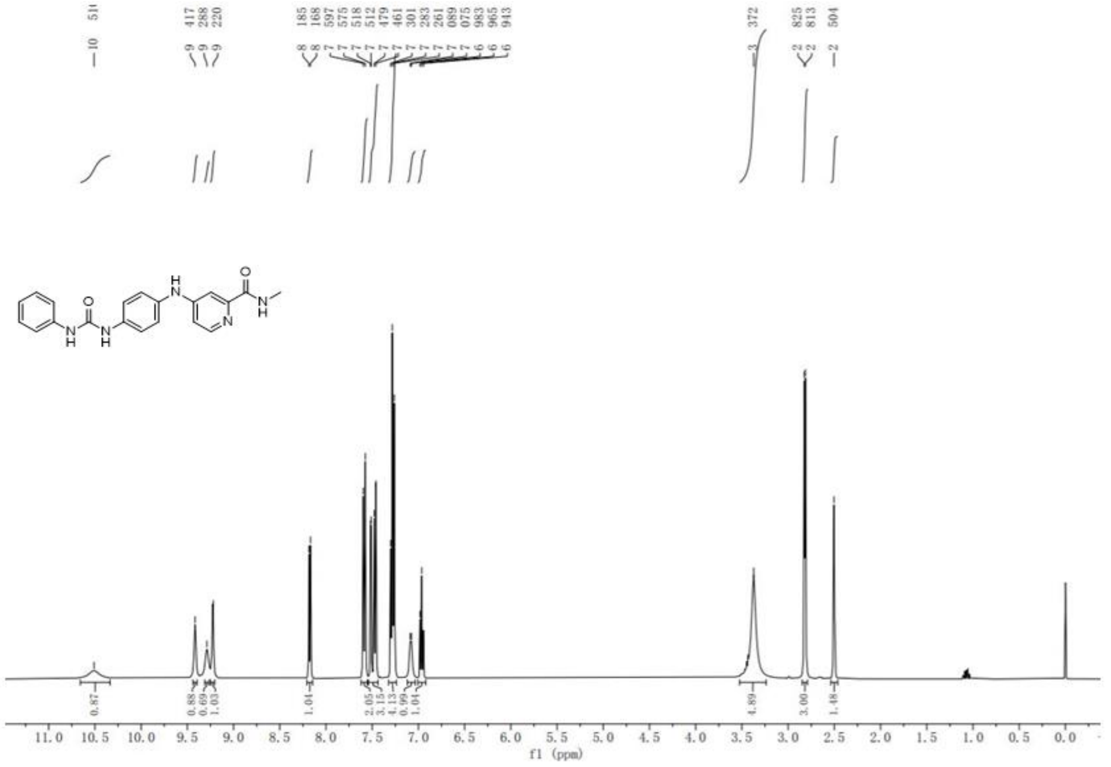

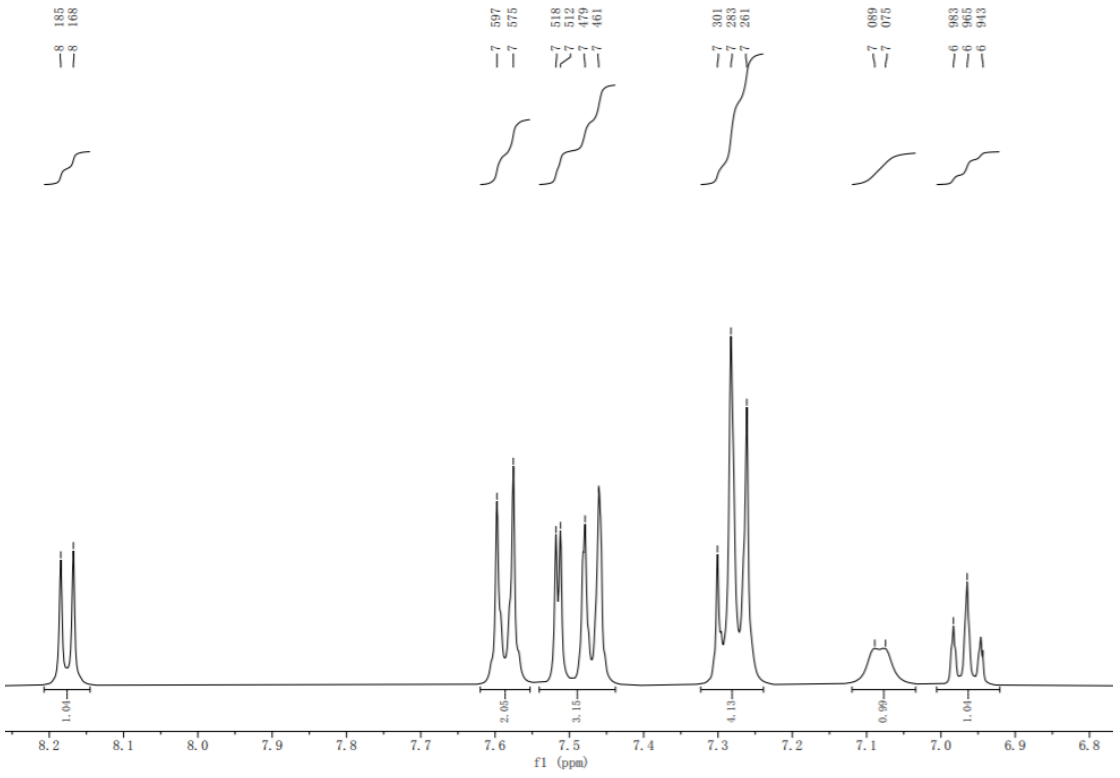

2.2 ^{1}H NMR of 5b

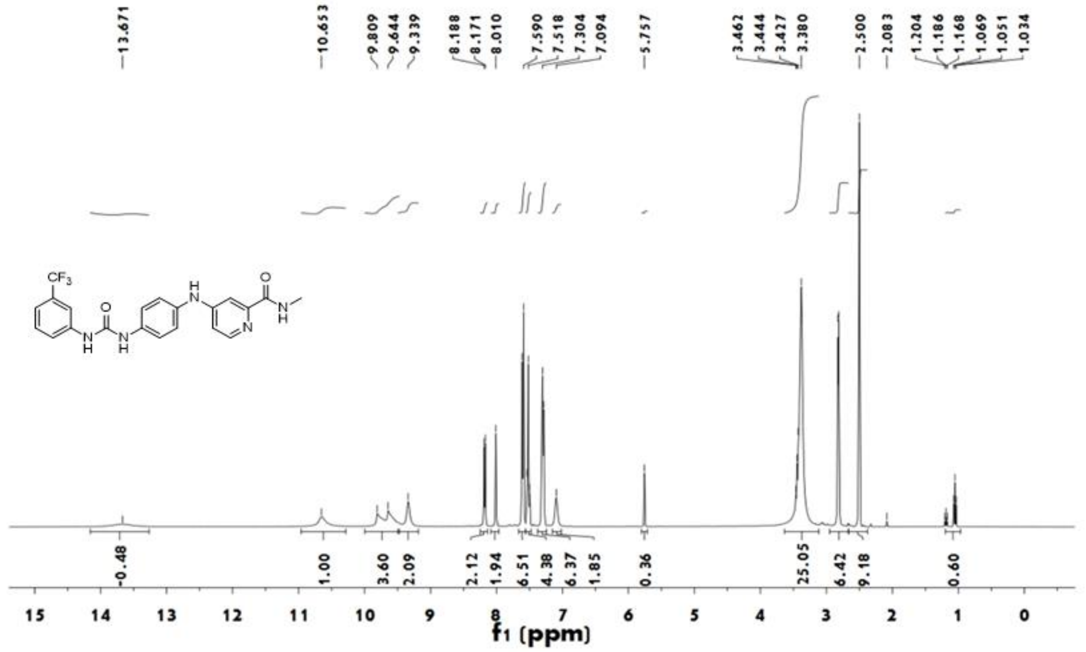

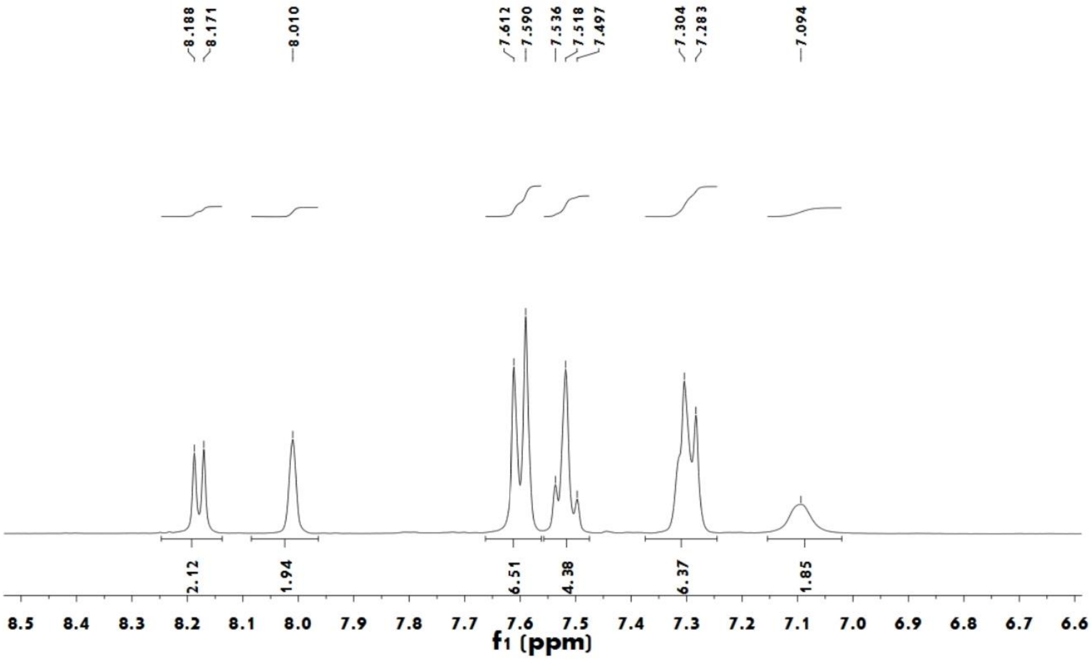

2.3 ^{1}H NMR of 5d

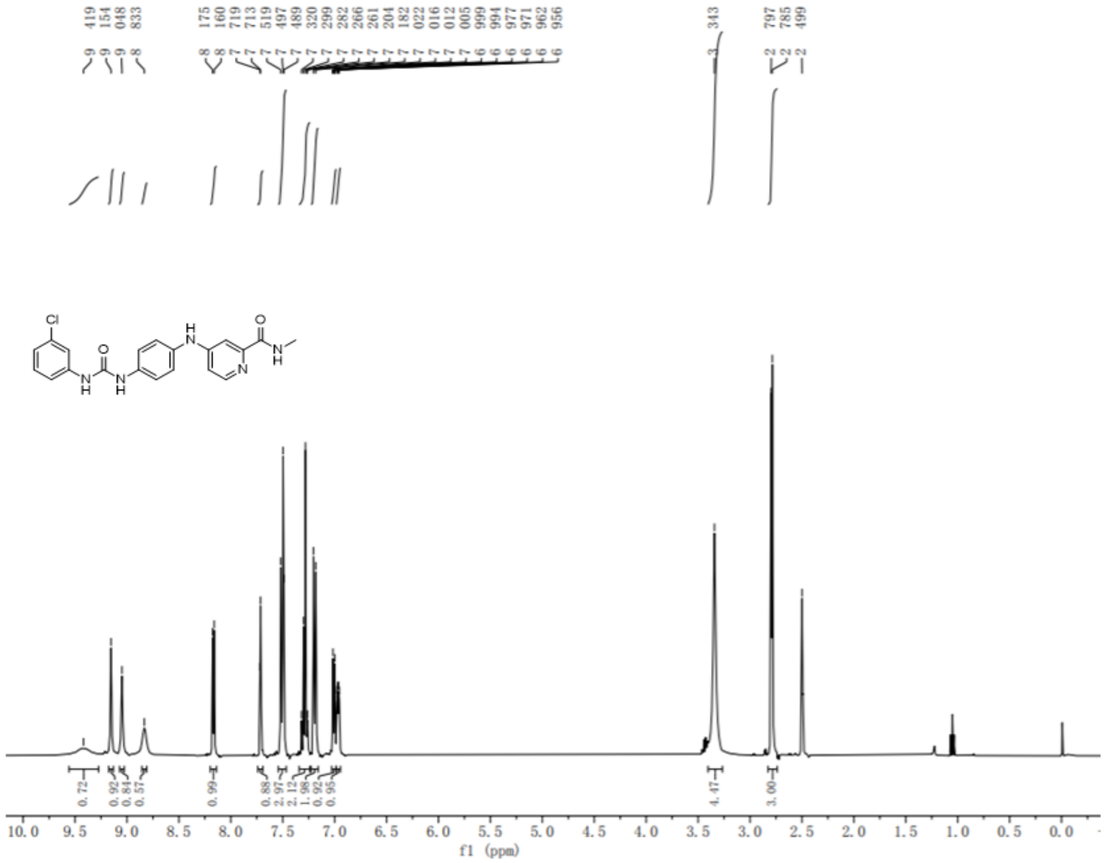

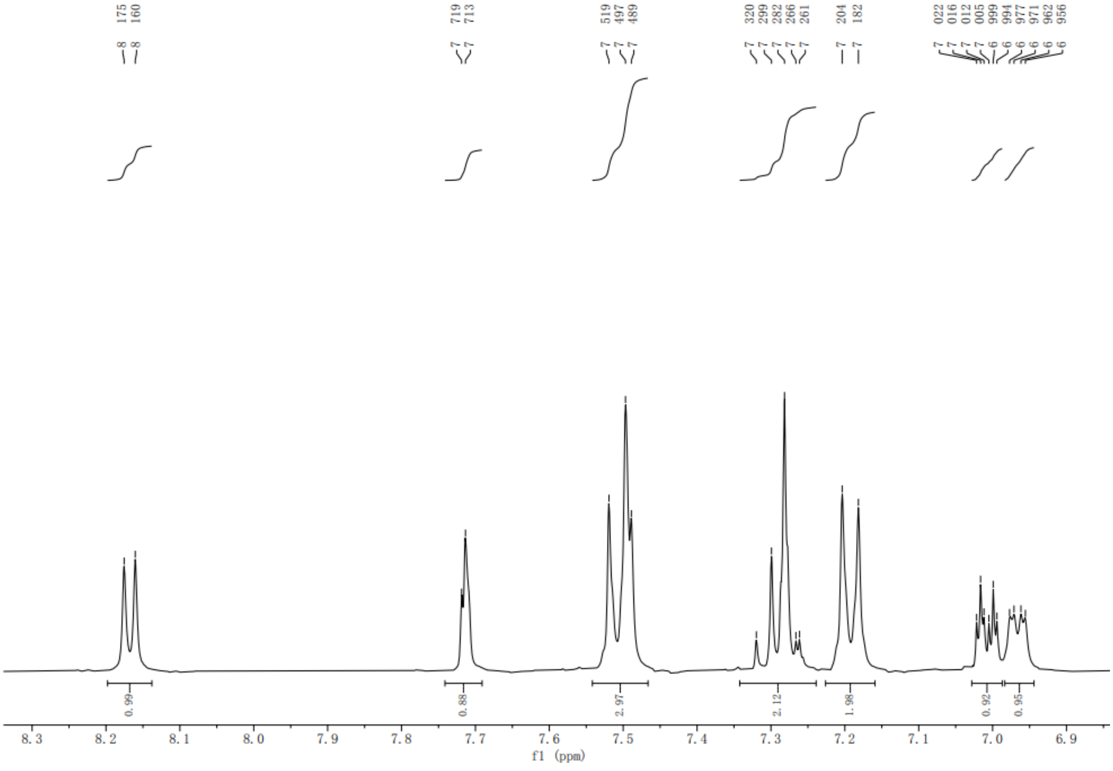

2.4 ^{1}H NMR of 5e

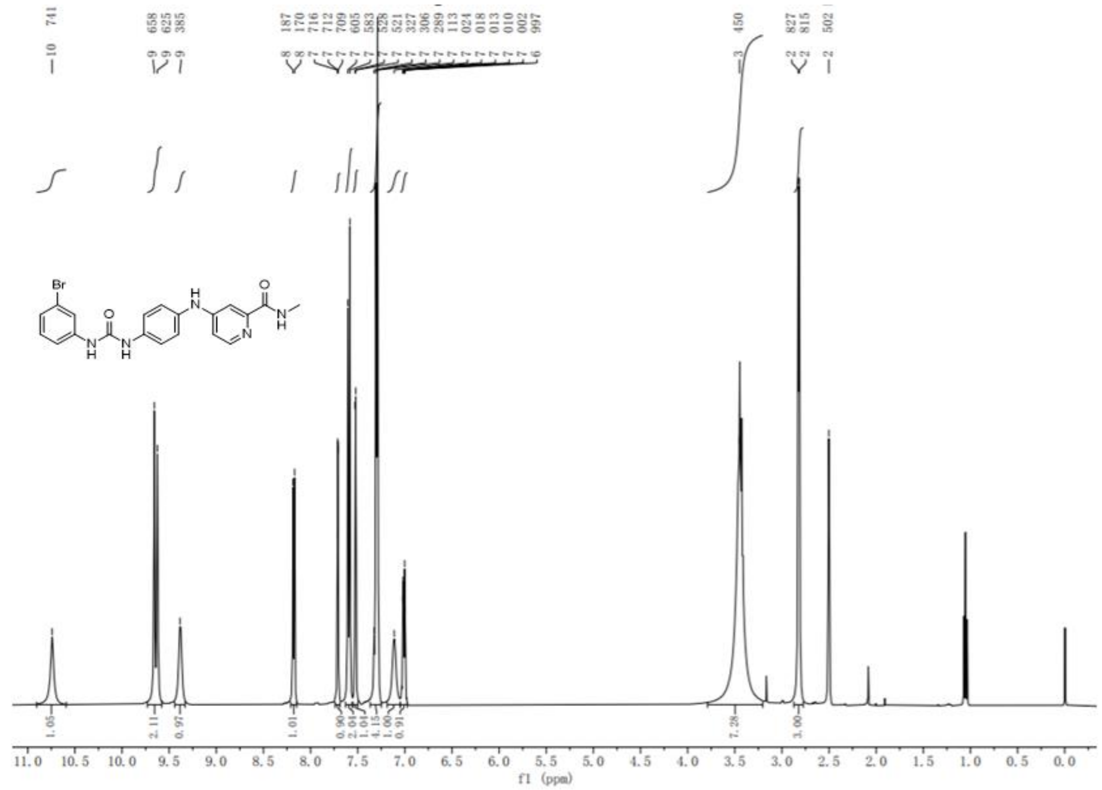

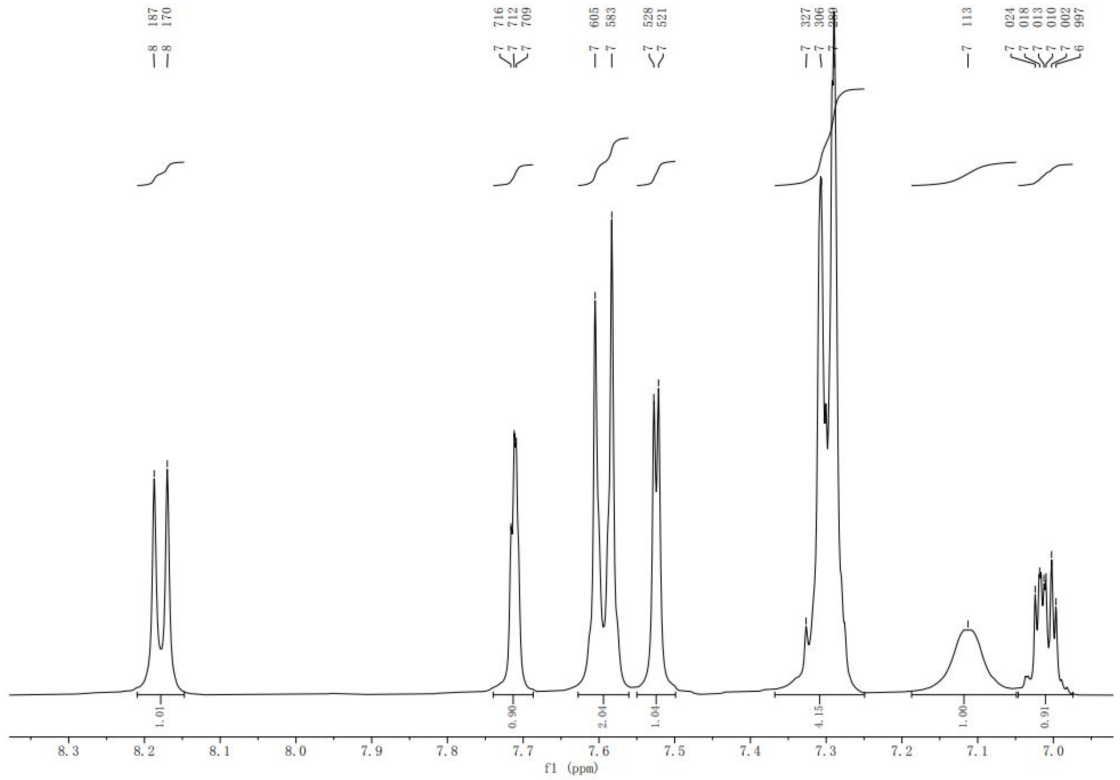

2.5 ^{1}H NMR of 5f

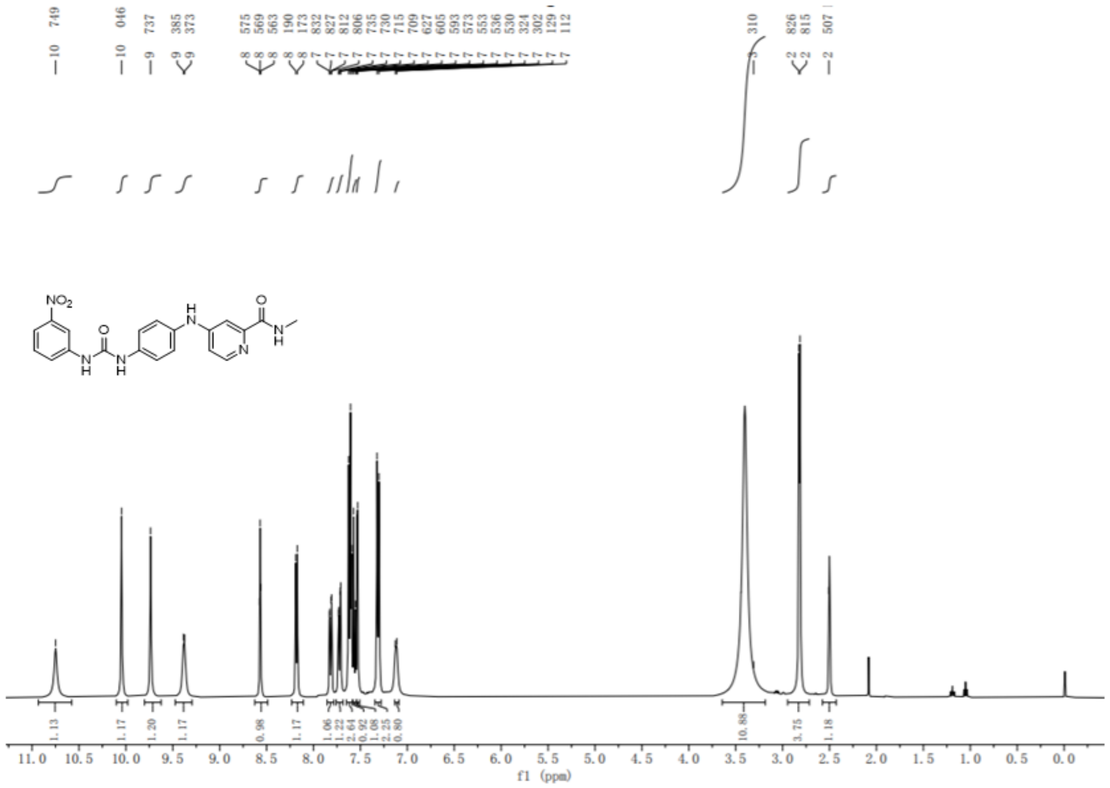

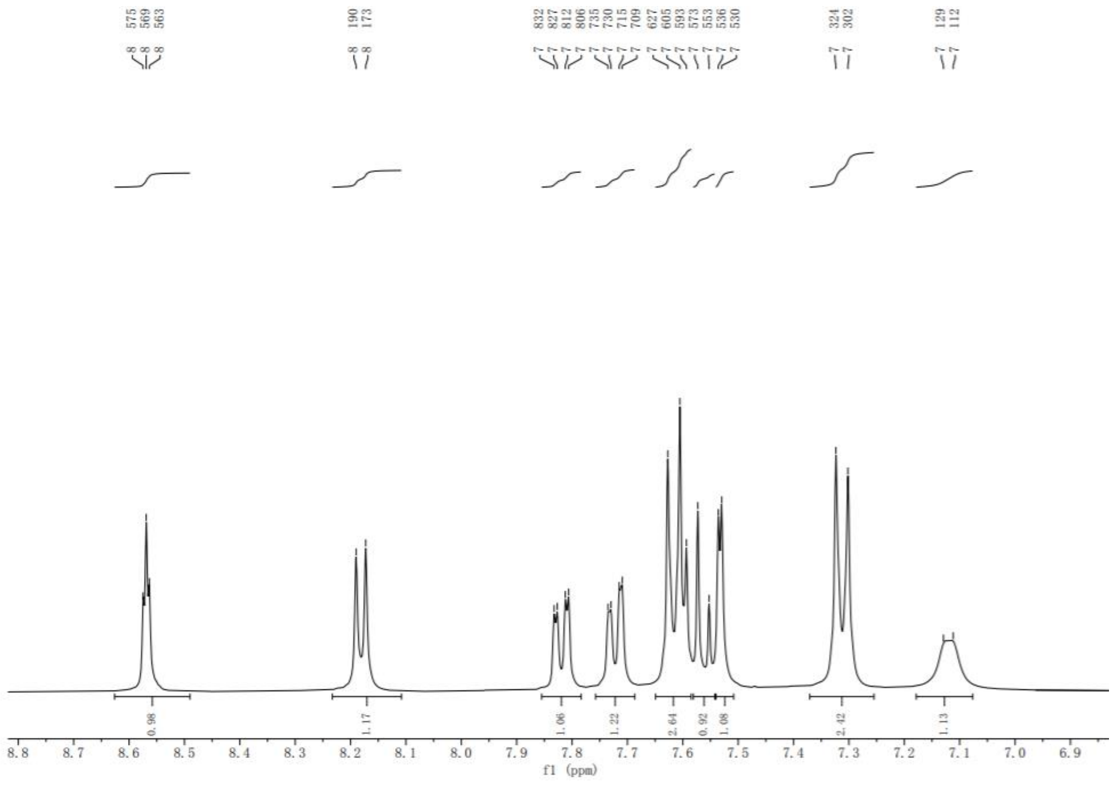

2.6 ^{1}H NMR of 5g

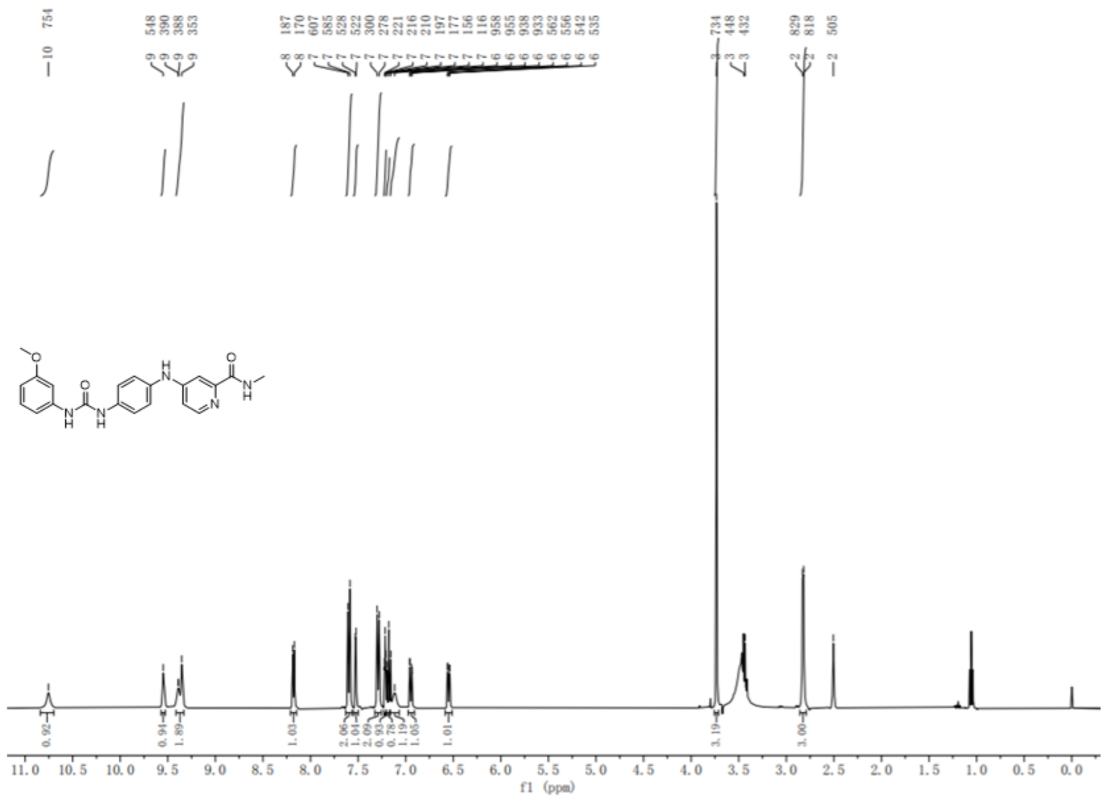

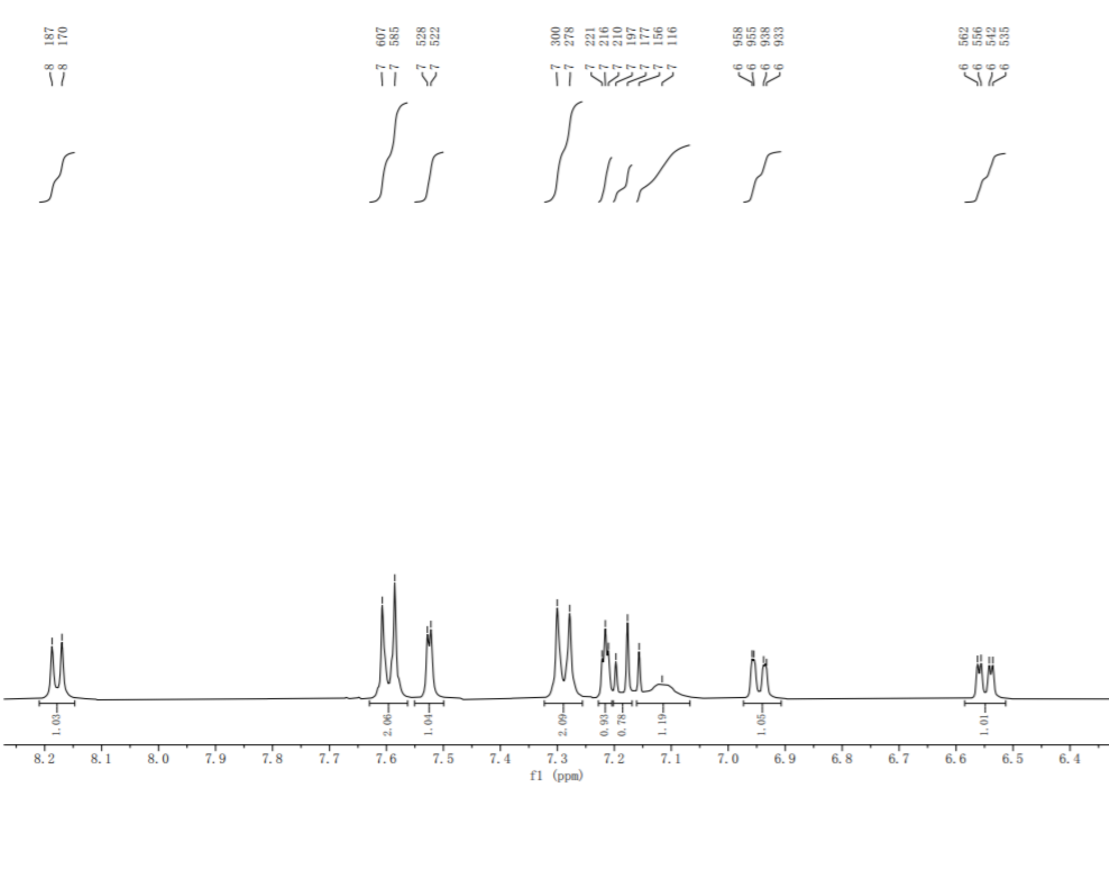

2.7 ^{1}H NMR of 5h

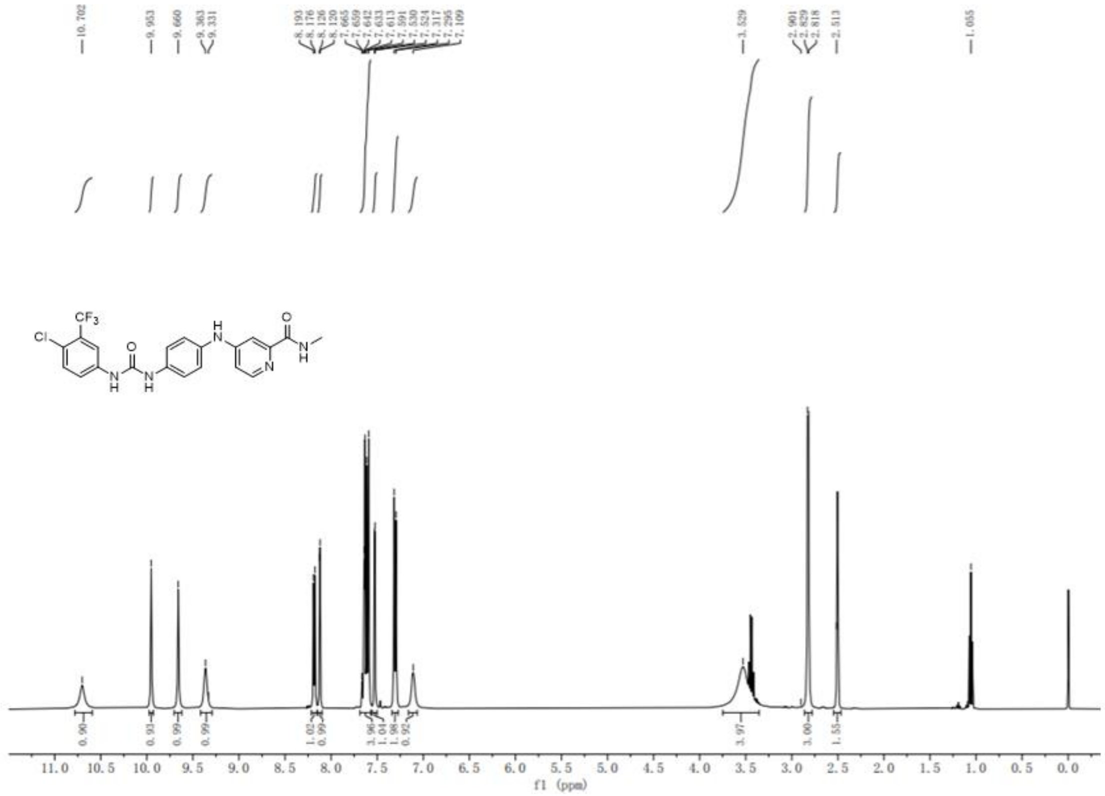

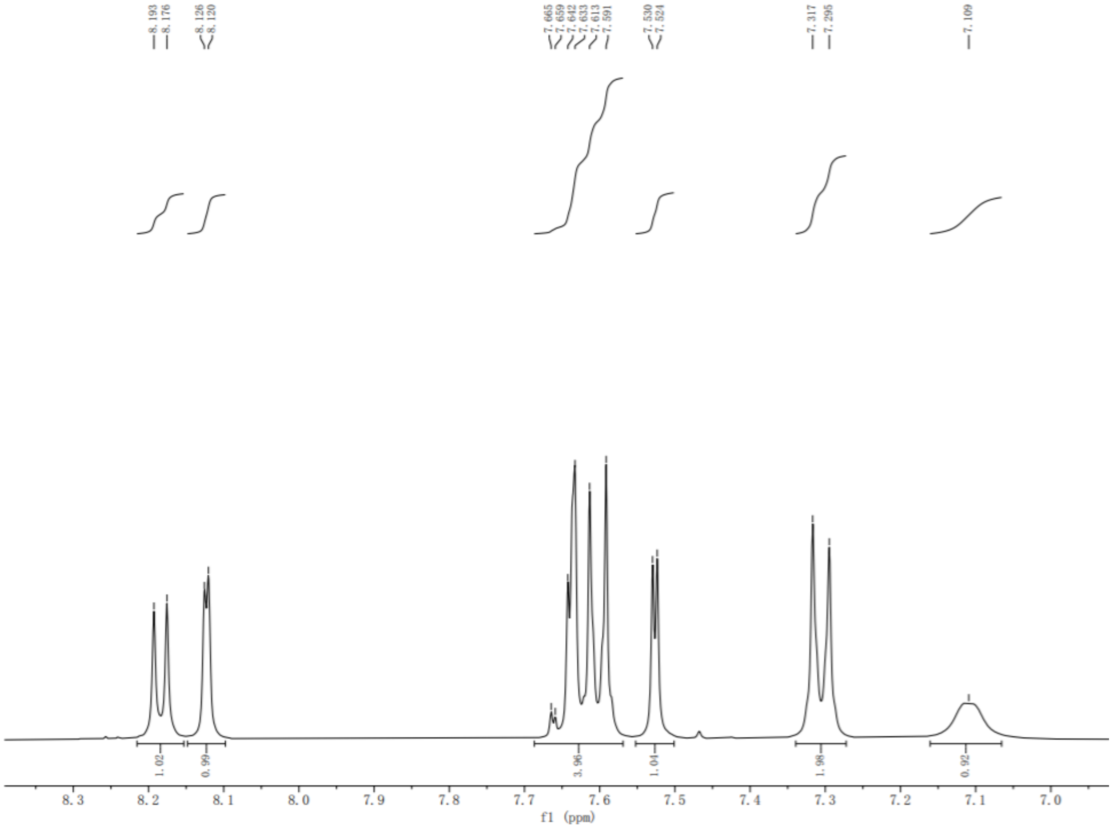

2.8 ^{1}H NMR of 5j

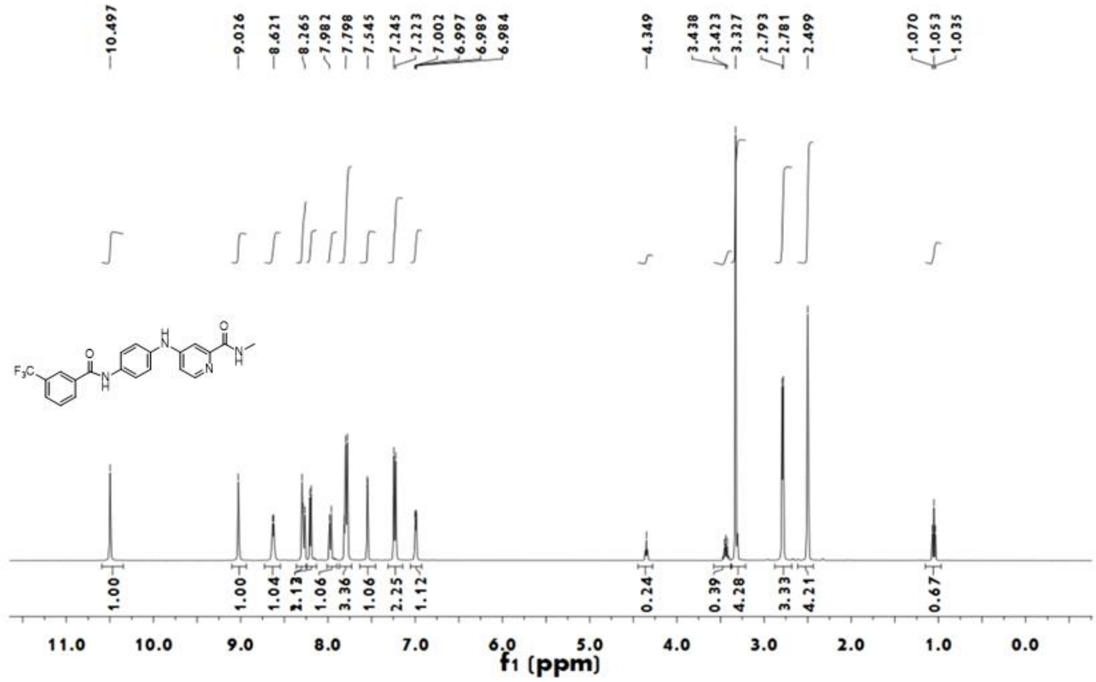

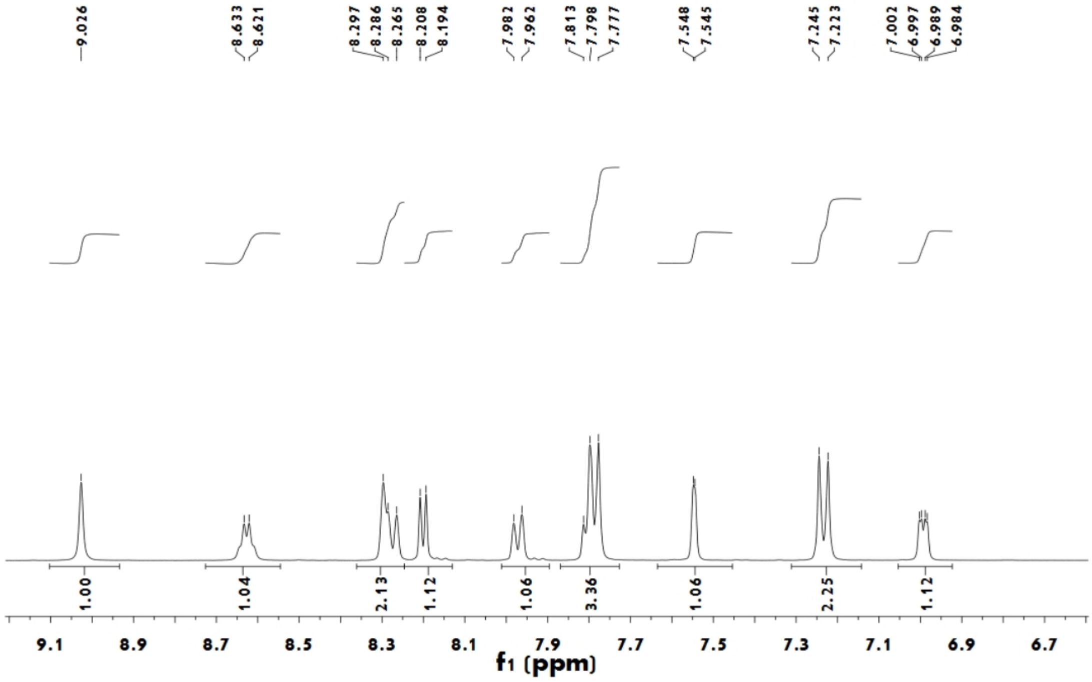

2.9 ^{1}H NMR of 5k

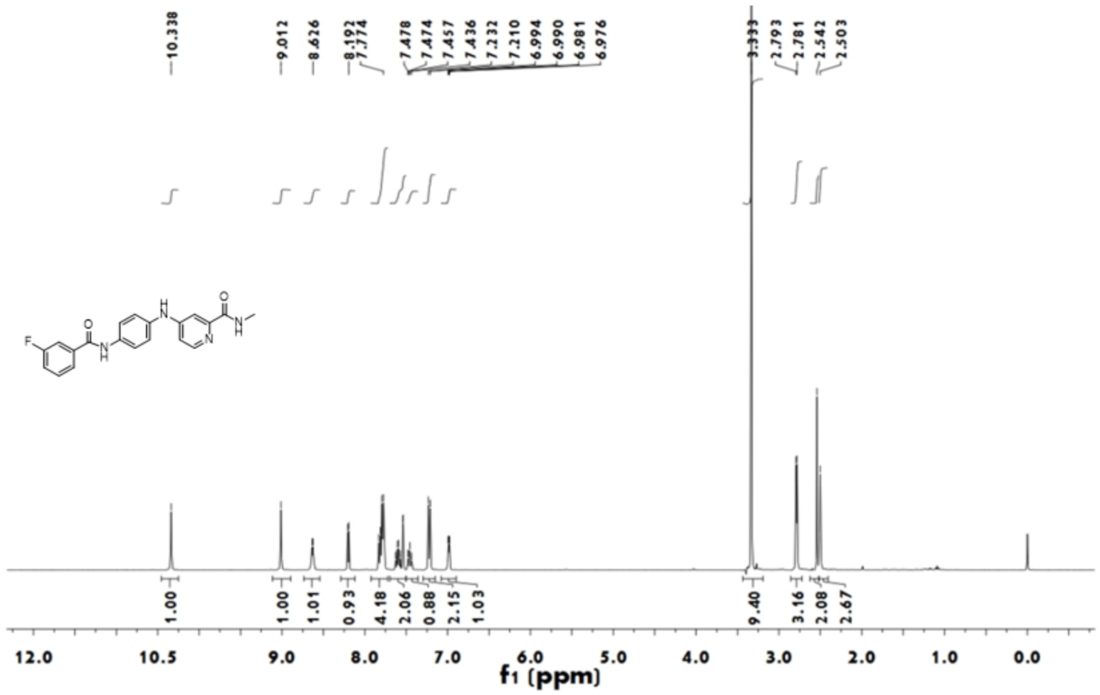

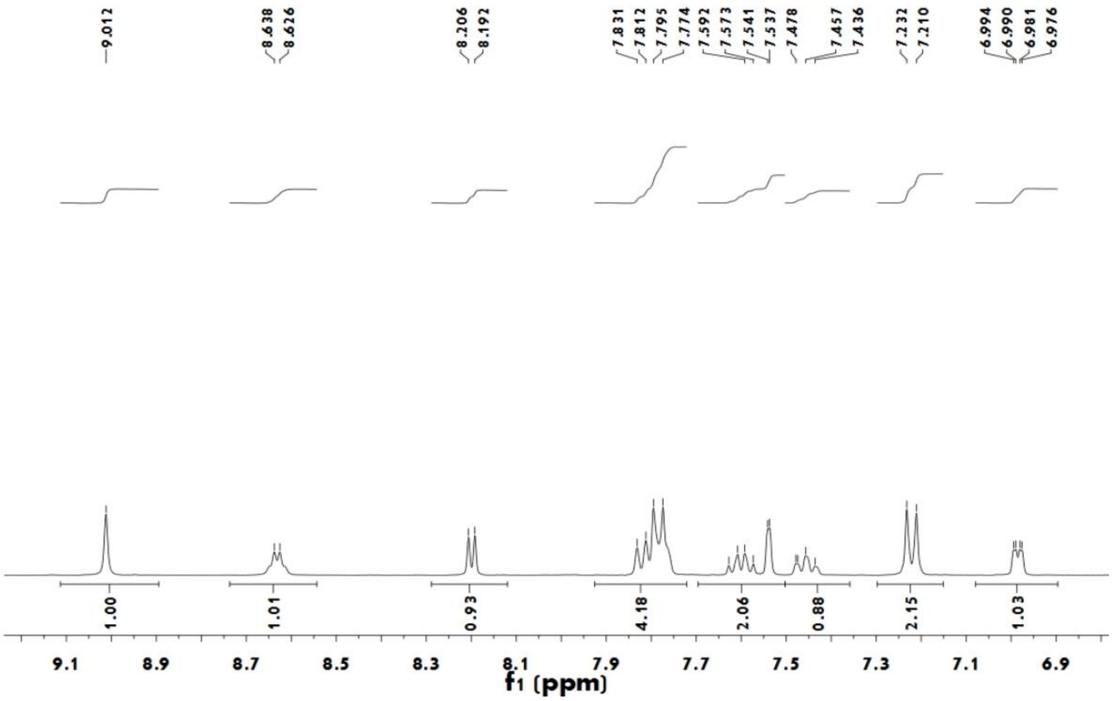

2.10 ^{1}H NMR of 5l

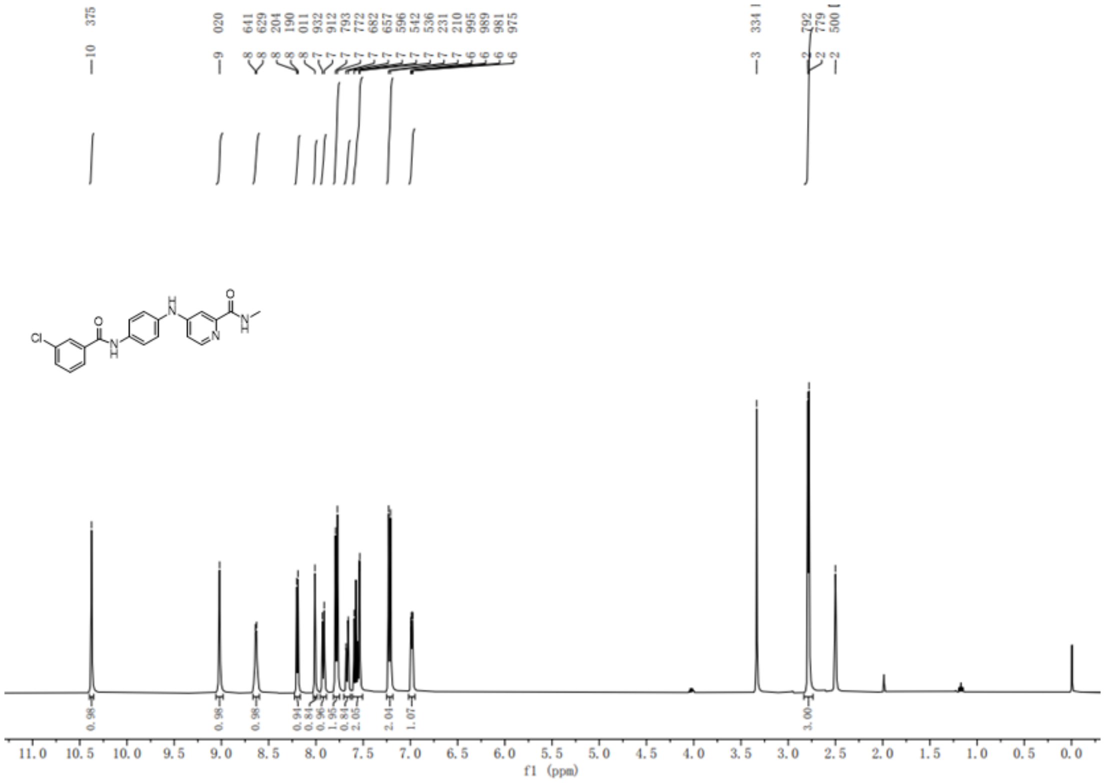

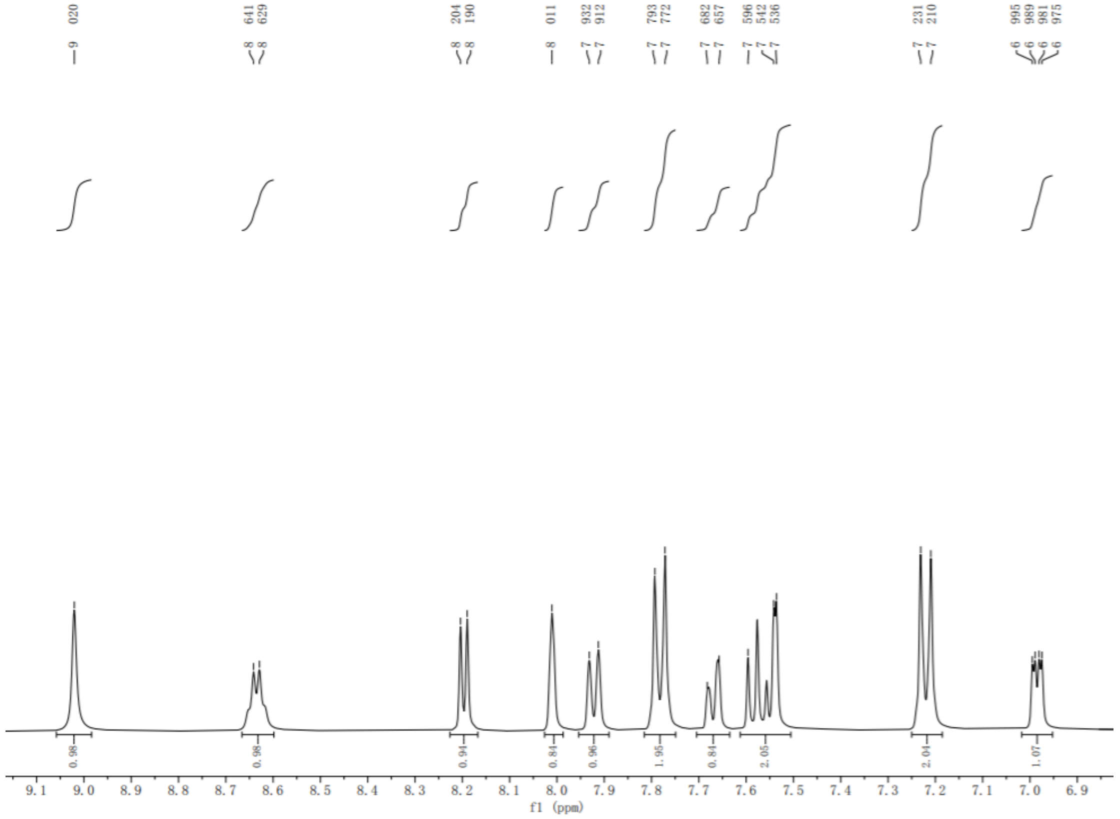

2.11 ¹H NMR of 5m

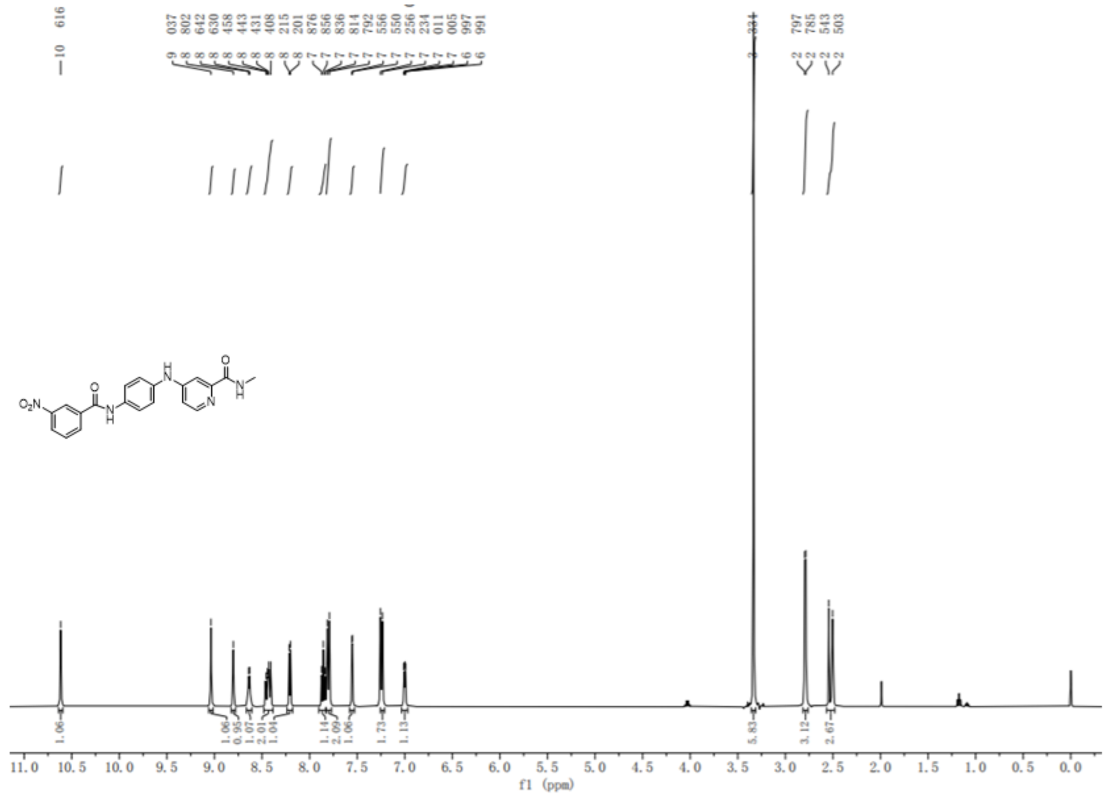

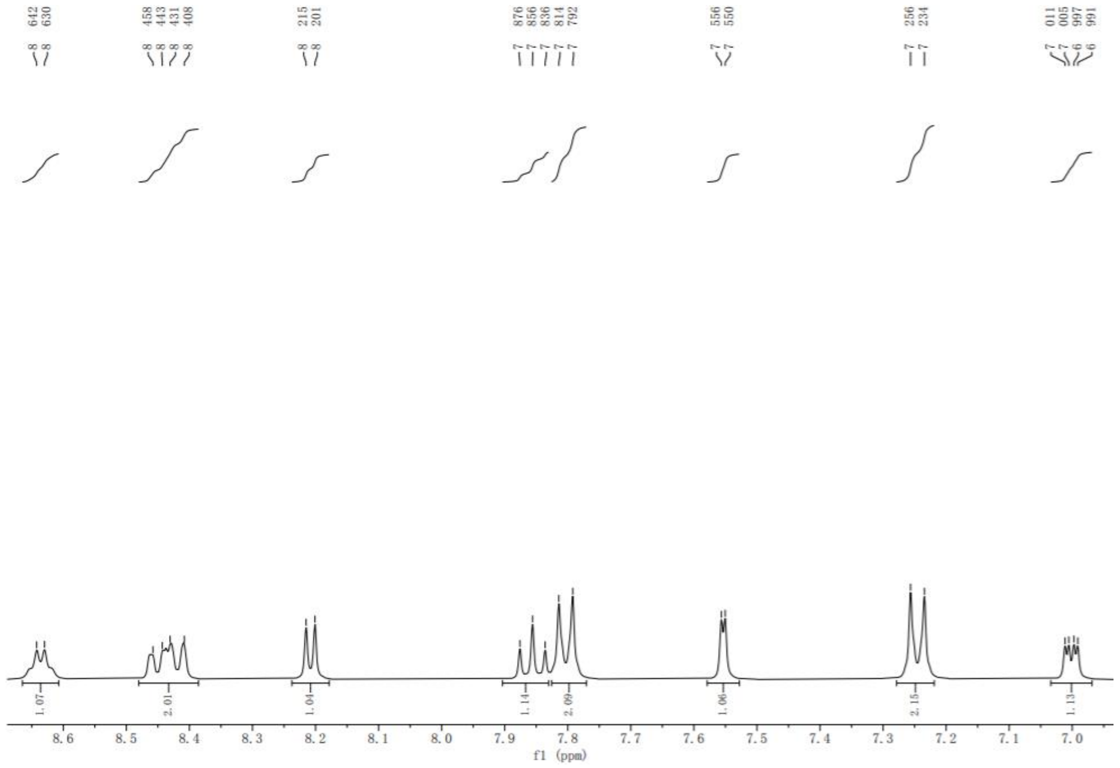

2.12 ^{1}H NMR of 5n

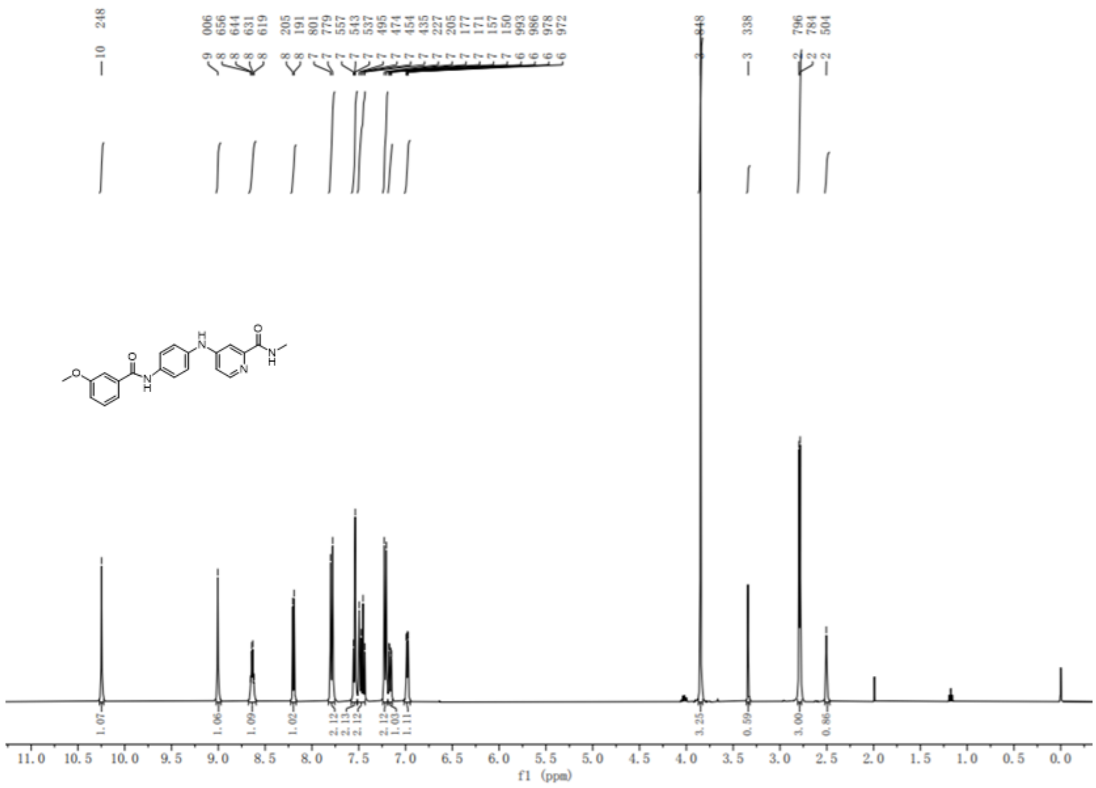

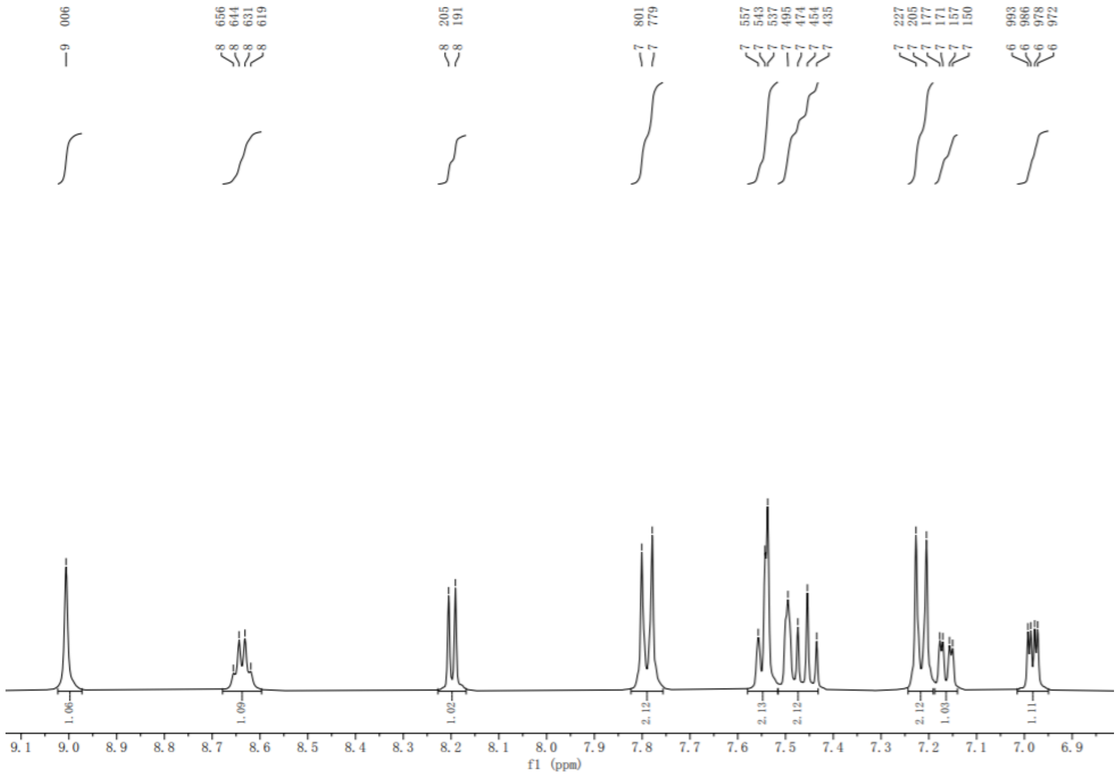

2.13 ¹H NMR of 5o

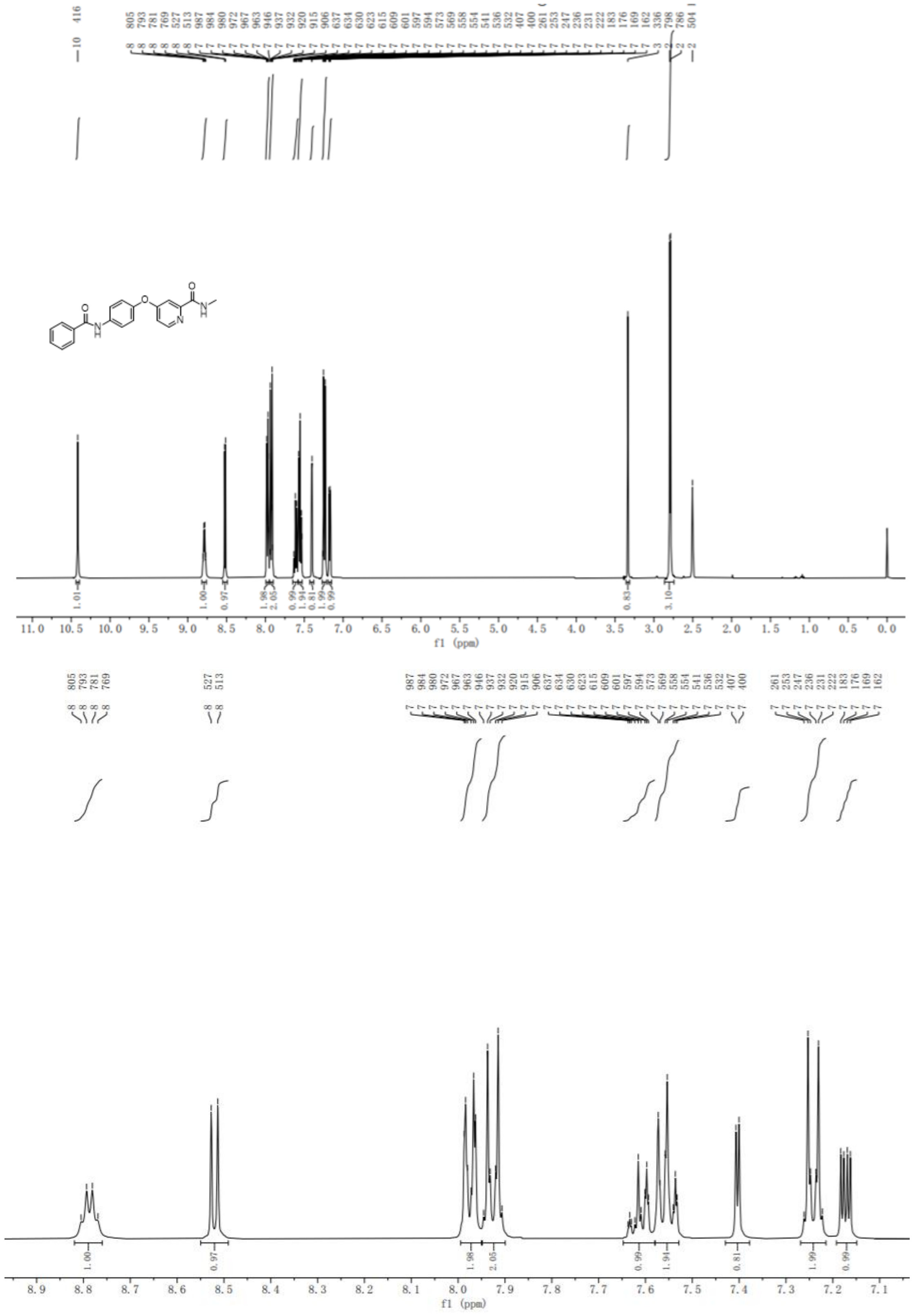

2.14 ^{1}H NMR of 5q

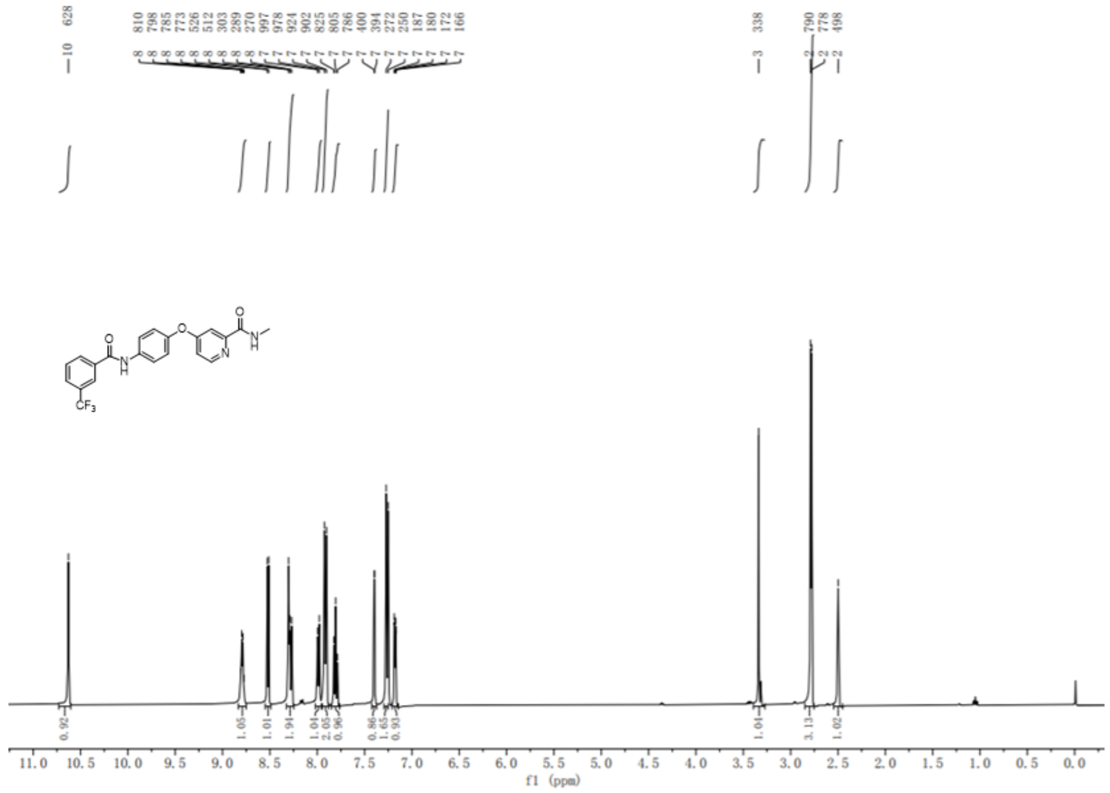

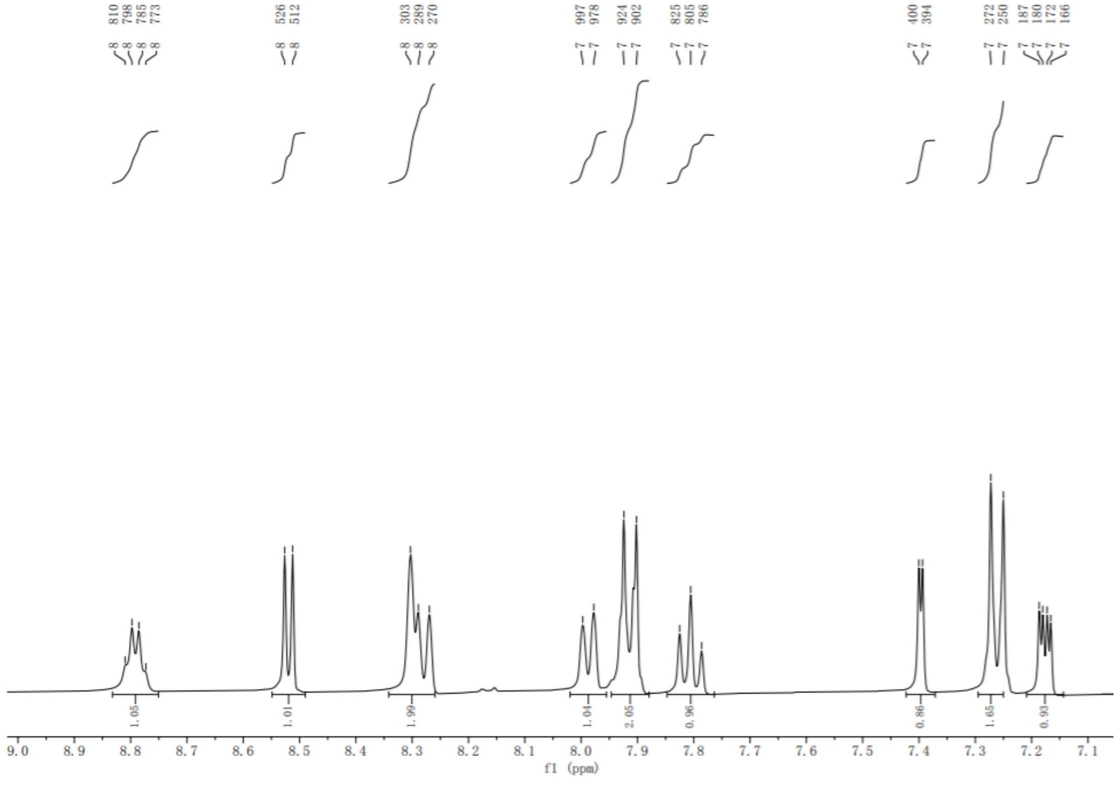

2.15 ^{1}H NMR of 5s

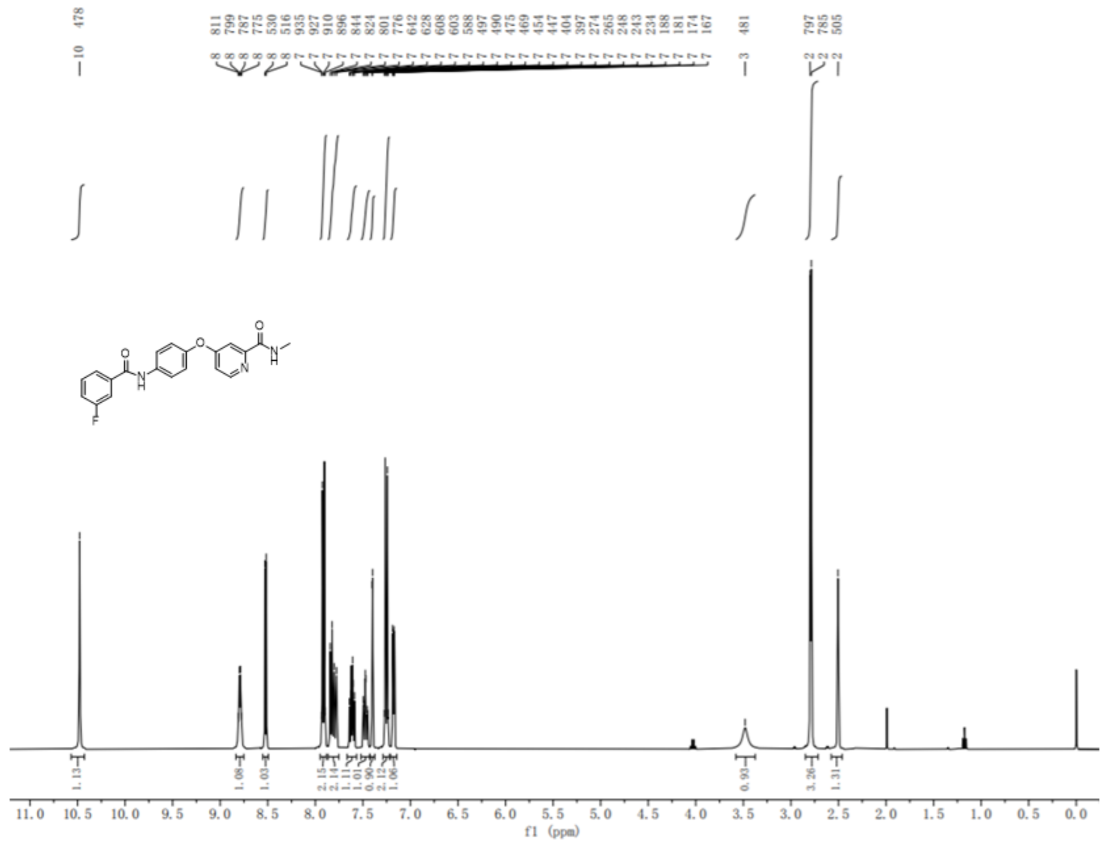

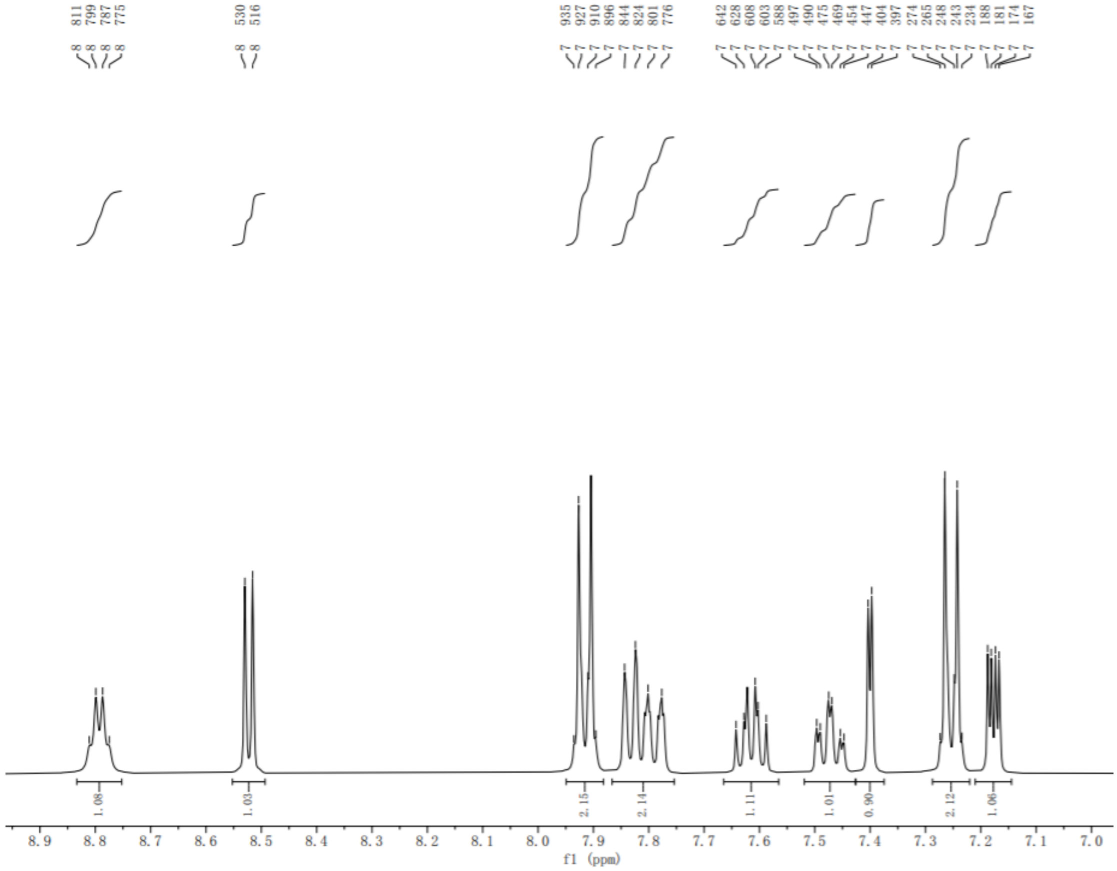

2.16 ^1H NMR of 5t

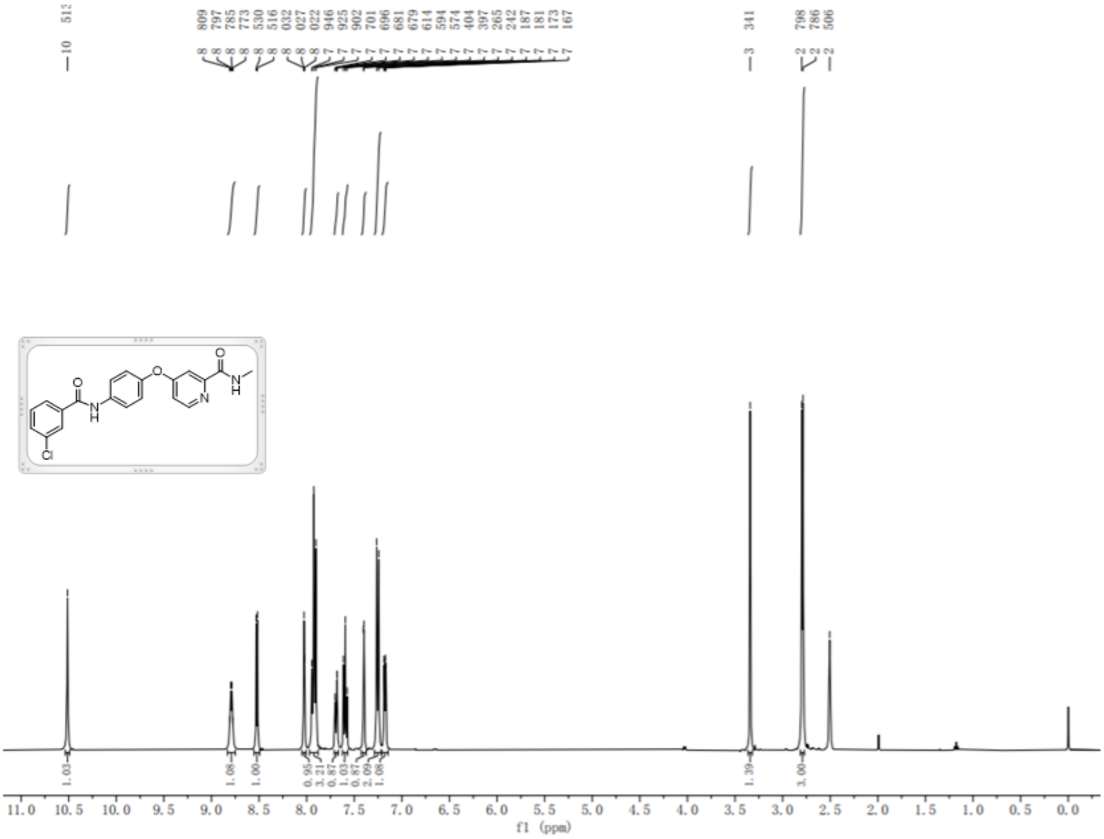

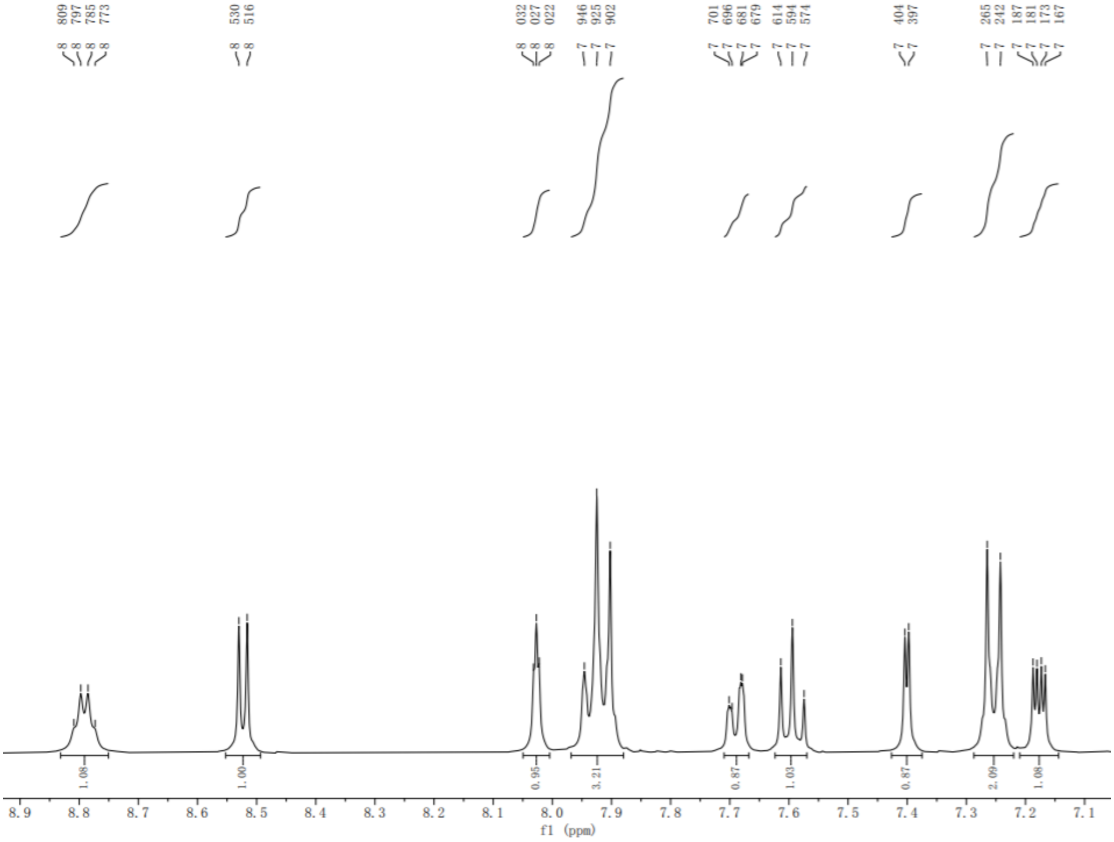

2.17 ^{1}H NMR of 5u

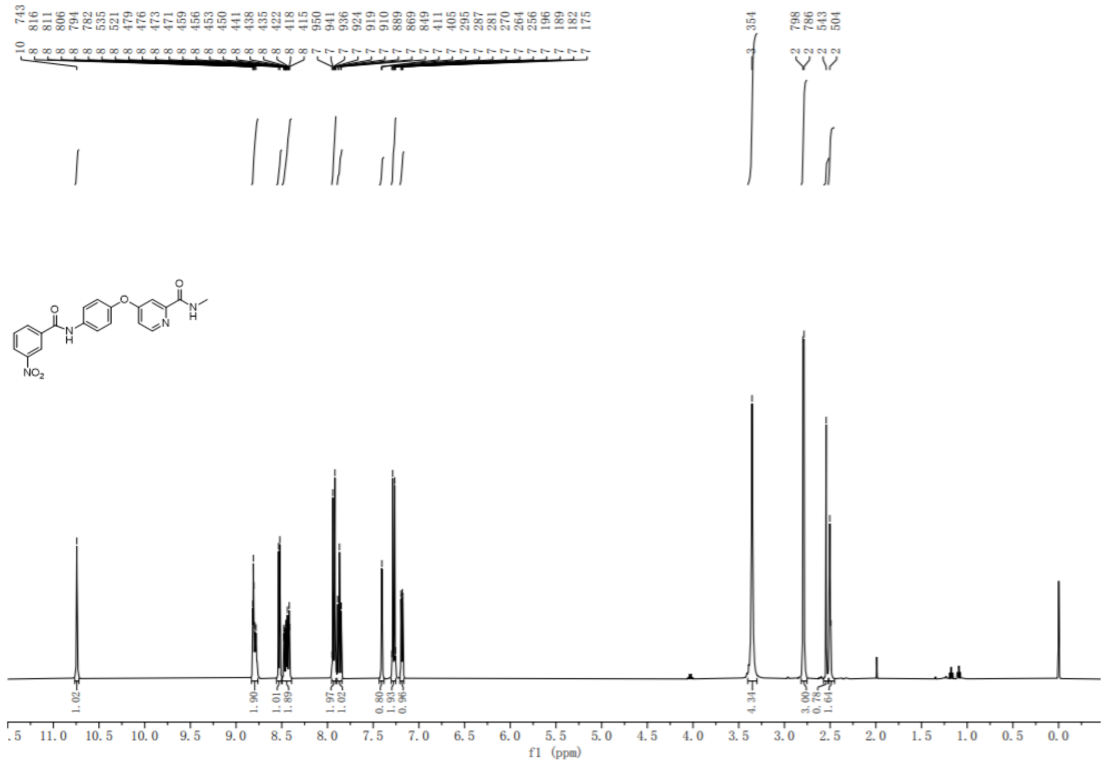

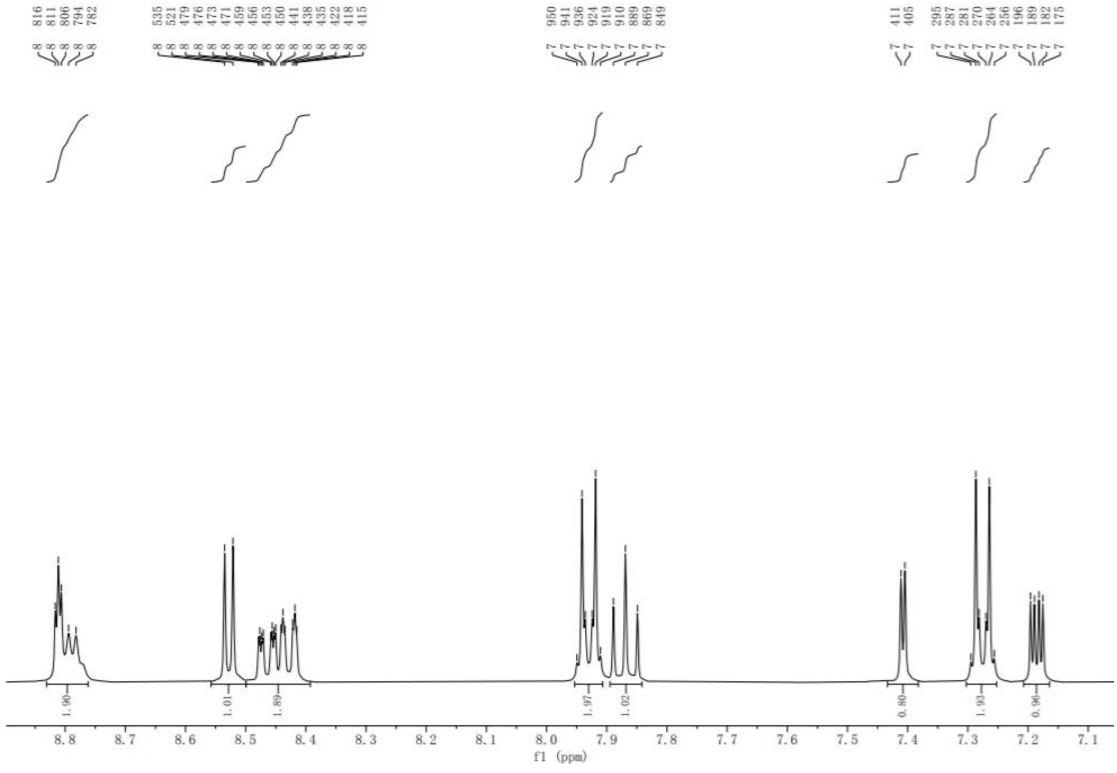

2.18 ^{1}H NMR of 5v

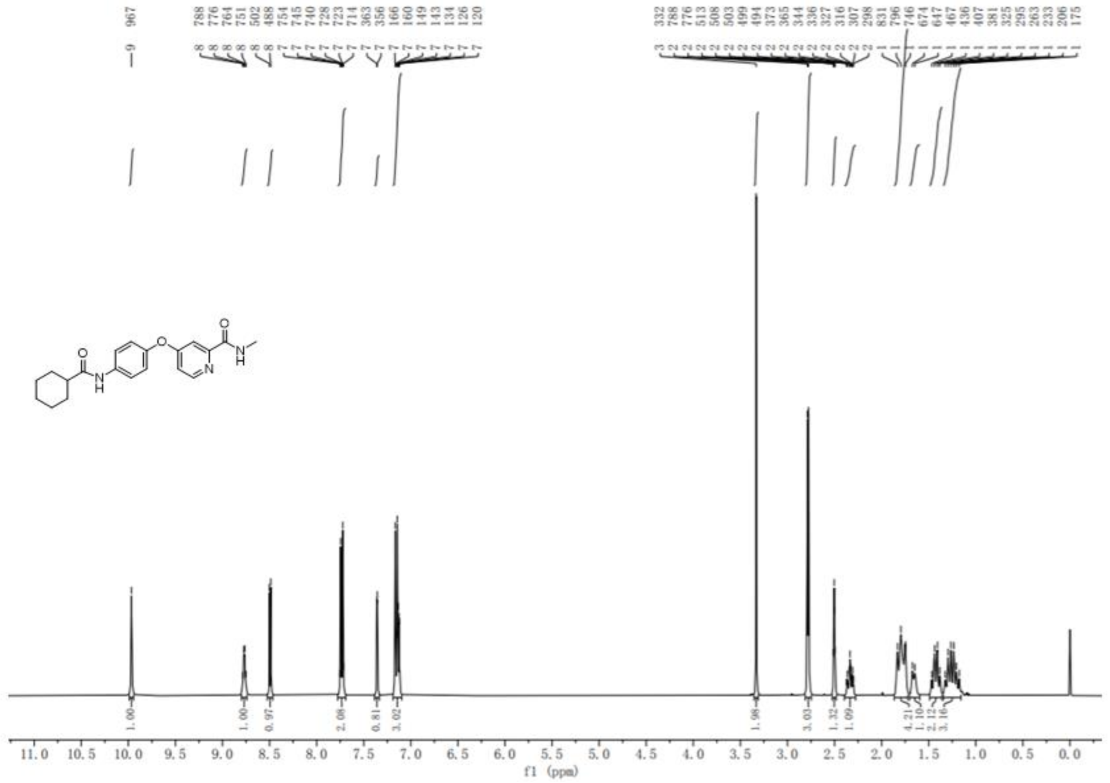

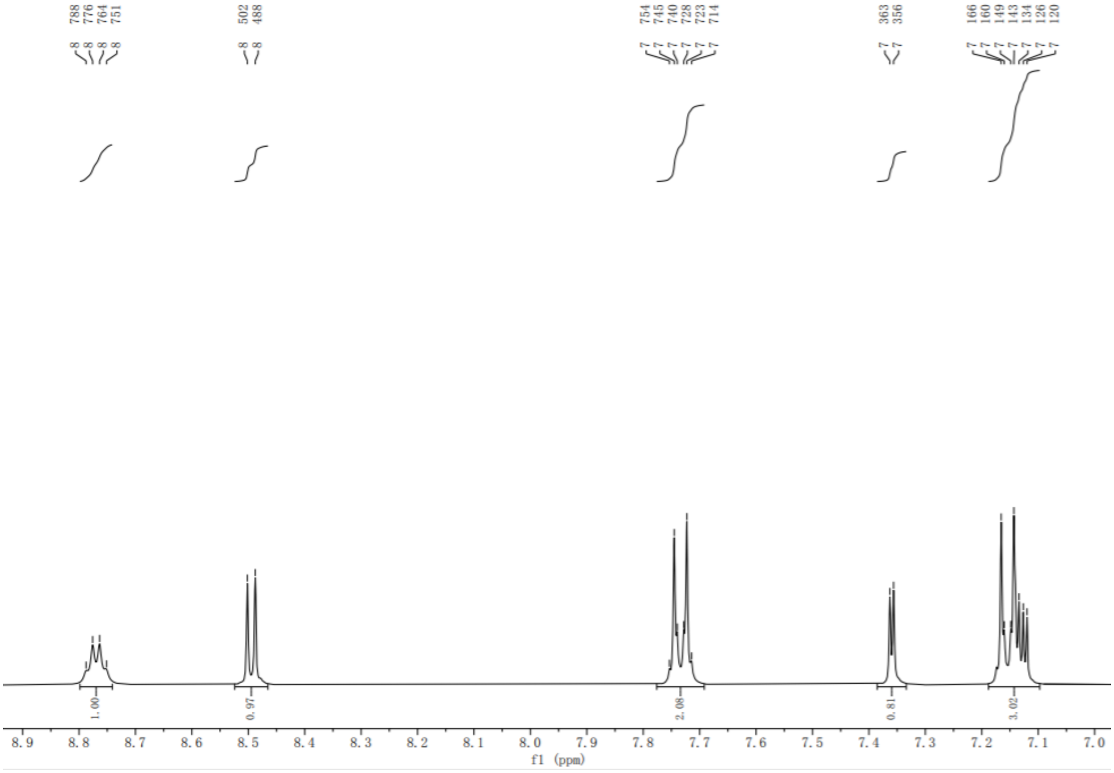

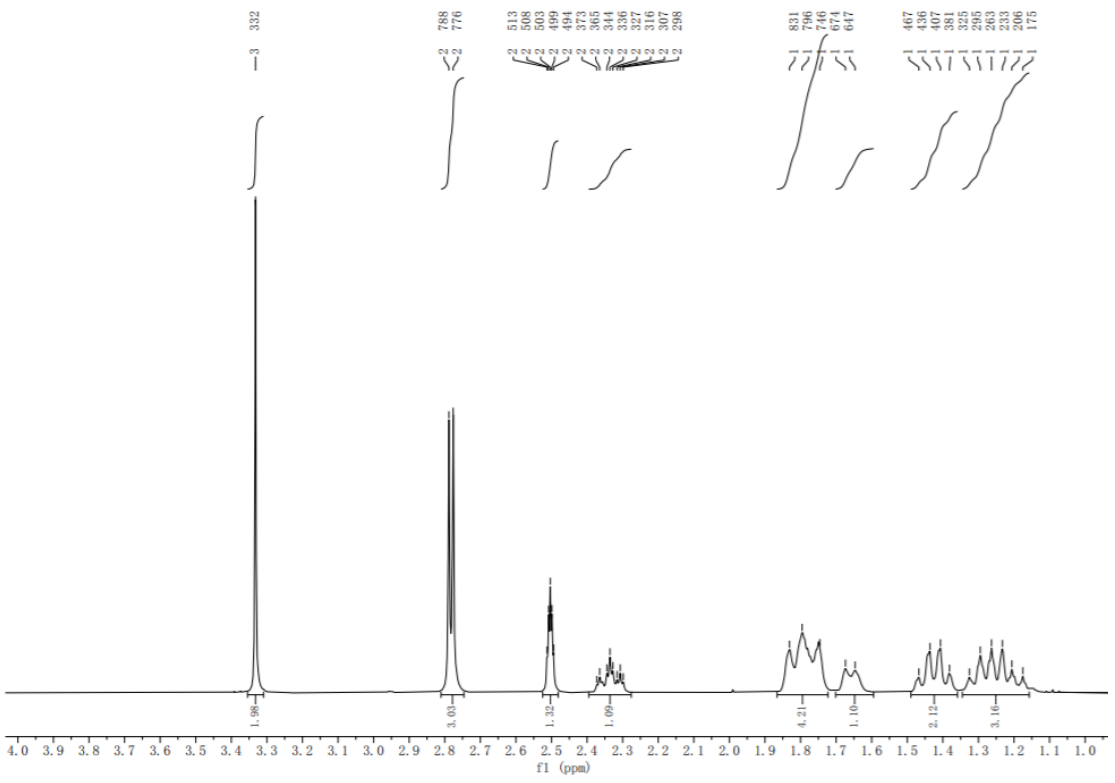
f1 (ppm)

2.19 ^{1}H NMR of 4a

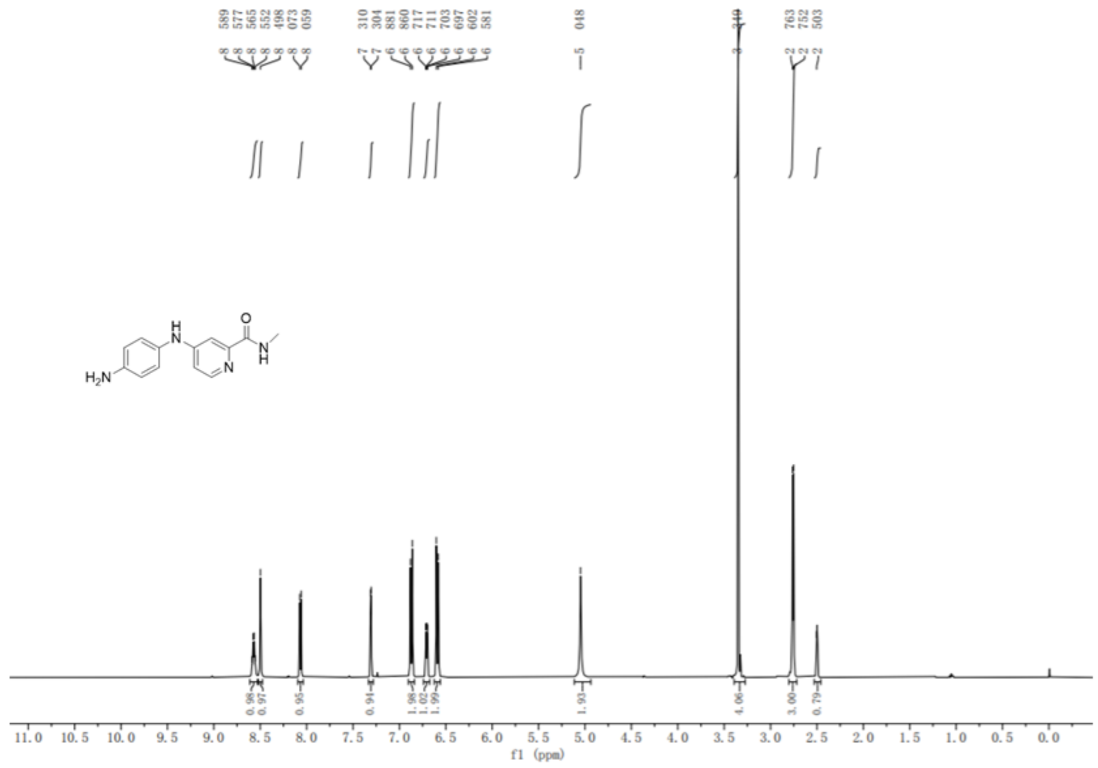

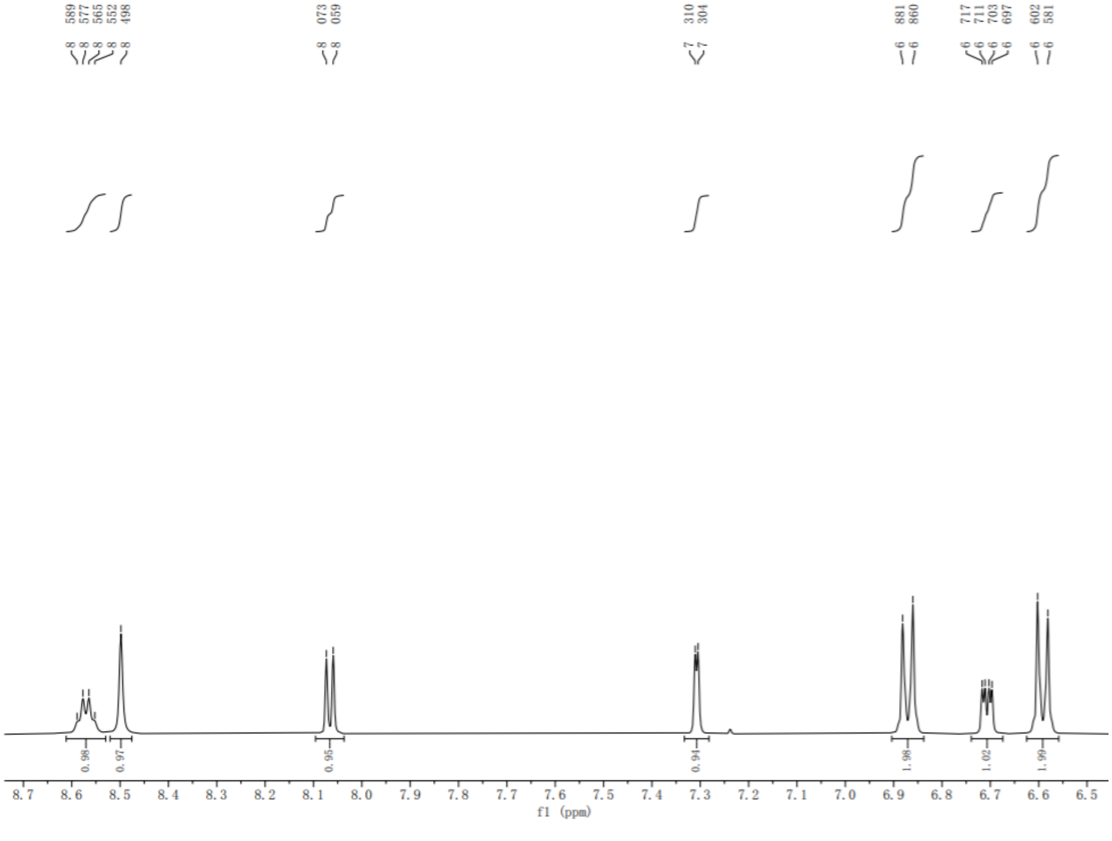

Supplement: Supplementary file 1 [file molecules-26-01150-s001.pdf]
